# Identification of multiple novel genetic mechanisms that regulate chilling tolerance in Arabidopsis

**DOI:** 10.3389/fpls.2022.1094462

**Published:** 2023-01-12

**Authors:** Dipak Kumar Sahoo, Chinmay Hegde, Madan K. Bhattacharyya

**Affiliations:** ^1^ Department of Agronomy, Iowa State University, Ames, IA, United States; ^2^ Department of Electrical and Computer Engineering, Iowa State University, Ames, IA, United States

**Keywords:** cold tolerance mechanisms, Arabidopsis, phenomics platform, mutatnt analyses, GWAS, NBS-LRR, abiotic stress

## Abstract

**Introduction:**

Cold stress adversely affects the growth and development of plants and limits the geographical distribution of many plant species. Accumulation of spontaneous mutations shapes the adaptation of plant species to diverse climatic conditions.

**Methods:**

The genome-wide association study of the phenotypic variation gathered by a newly designed phenomic platform with the over six millions single nucleotide polymorphic (SNP) loci distributed across the genomes of 417 Arabidopsis natural variants collected from various geographical regions revealed 33 candidate cold responsive genes.

**Results:**

Investigation of at least two independent insertion mutants for 29 genes identified 16 chilling tolerance genes governing diverse genetic mechanisms. Five of these genes encode novel leucine-rich repeat domain-containing proteins including three nucleotide-binding site-leucine-rich repeat (NBS-LRR) proteins. Among the 16 identified chilling tolerance genes, *ADS2* and *ACD6* are the only two chilling tolerance genes identified earlier.

**Discussion:**

The 12.5% overlap between the genes identified in this genome-wide association study (GWAS) of natural variants with those discovered previously through forward and reverse genetic approaches suggests that chilling tolerance is a complex physiological process governed by a large number of genetic mechanisms.

## Introduction

Globally, 13.4 billion hectares, or one-third of the total land area, is potentially suitable for arable agriculture. Unfortunately, because of abiotic stresses, only approximately one-ninth of the potentially arable land is ideal for crop production (Bruinsma, 2003). Severe weather conditions such as extreme cold, substantial and extended precipitation (Rosenzweig et al., 2002; Li et al., 2019b), hailstorms (Sánchez et al., 1996), and heatwaves and droughts (Ciais et al., 2005; van der Velde et al., 2010) limit agricultural productivity worldwide. Abiotic stresses affect the farming of existing crop species and act as a significant barrier to the introduction of new crops. A change in the expression levels of many genes allows the adaption of plant species to unique geographical regions. For example, an investigation of Arabidopsis ecotypes collected from broad geographical regions has revealed genes essential for adaptation (Fournier-Level et al., 2011; Hancock et al., 2011).

Cold stress adversely affects plant growth and development and restricts the geographical distribution of many plant species. Plants are classified as either chilling (0-15°C) or freezing (< 0°C) tolerant. These two classes are not mutually exclusive, as chilling-tolerant plants in a temperate climate can induce their freezing resilience after exposure to chilling or non-freezing temperatures during cold acclimation (Lyons and Breidenbach, 1981). Cold acclimation in plants is linked to biochemical and physiological changes resulting from altered gene expression, bio-membrane lipid composition, and accumulation of small molecules (Thomashow, 1998; Yamaguchi-Shinozaki and Shinozaki, 2006; S. Sanghera et al., 2011). Cold tolerance is a multifaceted trait linked to numerous cell compartments and metabolic pathways regulated by reprogrammed gene expression (Hannah et al., 2005). Plants from tropical and subtropical regions lack cold acclimation machinery and are sensitive to chilling stress. The molecular basis of cold acclimation and acquired freezing tolerance has been investigated extensively in plants like Arabidopsis and winter cereals. The forward and reverse genetics studies in Arabidopsis have identified several players involved in cold tolerance (Hannah et al., 2006; Guo et al., 2021). Many candidate genes and genetic loci have been identified in cereals through genome-wide association studies (GWAS) (Rathan et al., 2022). This study was undertaken to complement the effort of ongoing cold tolerance studies in plants and identify any possible novel genetic cold tolerance mechanisms by conducting GWAS of natural variants and insertion mutant analyses in Arabidopsis.

Arabidopsis is an ideal model plant for dissecting genetic pathways involved in combating environmental stresses. The 1,001 Arabidopsis Genomes Project initiative led to the resequencing of 1,135 natural inbred lines collected from the native Eurasian/North African range and the recently colonized North America (Alonso-Blanco et al., 2016). Genome-wide association studies (GWAS) of these natural variants adapted to three diverse ecological environments are expected to facilitate the identification of genetic mechanisms for adapting Arabidopsis to distinct climatic conditions. Earlier, GWAS in a limited number of natural variants of Arabidopsis revealed candidate genetic loci for adaptation (Fournier-Level et al., 2011; Hancock et al., 2011). GWAS of natural Arabidopsis variants can identify candidate genes for physiological functions. The function of such candidate genes can then be validated by studying knockout mutants for these genes. There are over 260,000 individual mutant lines in the Arabidopsis community allowing identification of knockout and knockdown mutants for most of the 29,454 predicted protein-coding Arabidopsis genes (Alonso et al., 2003; O’Malley and Ecker, 2010; O’Malley et al., 2015). Recently, digital photo-based objective phenotyping for this model plant has also been established for high-throughput phenomics studies (Manacorda and Asurmendi, 2018; Vasseur et al., 2018).

In this study, we have (i) developed a high-throughput digital photo-based objective phenotyping method for rosette leaves of Arabidopsis seedlings; (ii) collected responses of 417 resequenced diverse Arabidopsis natural variants to prolonged chilling temperature using this phenotyping system; (iii) conducted GWAS to identify candidate chilling tolerance genes; and (iv) validated the functions of individual candidate chilling tolerance genes by studying at least two independent Arabidopsis insertion mutants for each of the genes.

We identified 33 candidate genes involved in chilling tolerance. Investigation of at least two insertion mutants for each of 25 of these genes revealed 16 chilling tolerance genes. Surprisingly only two of these genes, *ADS2* encoding an acyl-lipid desaturase and *ACD6* encoding a novel ankyrin protein termed accelerated cell death 6, were previously identified as the cold tolerance genes (Lu et al., 2009; Chen and Thelen, 2013). Five LRR domain-containing genes were also identified, including three novel NB-LRR genes with no similarity to previously identified NB-LRR cold-stress-related genes. The identified genes include the ones involved in (i) lipid metabolism (*ADS2*), (ii) biotic stress-related genes (*NB-ARC LRR*, *TIR-NB-LRR*, *AtRLP39*, *PER72*, LRR protein kinase), (iii) ubiquitin and autophagy-dependent degradation pathway, (iv) proteolysis (*EDA41*), (v) vesicle transport and protein targeting pathway (*AtSYP112*), (vi) transcriptional regulation (*HMGB6*, stress-associated protein 7), and (vii) an abiotic stress-related heat stress gene (DNAJ heat shock N-terminal domain-containing protein). Our results suggest that chilling tolerance is a complex physiological process governed by many genetic mechanisms.

## Materials and methods

### Analyses of phenotypes

Seeds of 417 Arabidopsis (*Arabidopsis thaliana*) accessions (Alonso-Blanco et al., 2016) (**Table S1**) originating from diverse ecological regions (**Figure S1A**) were obtained from the Arabidopsis Biological Research Center (ABRC), Ohio State University, Columbus, OH, USA. To facilitate sowing of a similar number of seeds among replications, we compared two types of agaroses (standard agarose and low melting NuSieve agarose) at several concentrations (0.6%, 0.45%, 0.3%, 0.15%, and 0.1%); and 0.1% (w/v) NuSieve Agarose was found to be the best medium for keeping the seeds suspended for an extended period of time. Seeds were surface sterilized with 95% ethanol prior to sowing. To break the seed dormancy, the seeds were stratified in the agarose medium at 4°C in the dark for five days. After stratification, approximately 15 seeds from each ecotype were sown in individual cells of Plug Tray-288 (Growers Supply, IA, USA) filled with soil (Sungro Horticulture Professional Growing Mix, Hummert International, MO, USA).

Seeds of T-DNA and transposon insertion mutants for candidate cold stress-related genes identified by GWAS were obtained from the Arabidopsis Biological Resource Center (https://abrc.osu.edu) (**Table S1**) and propagated directly under optimal greenhouse conditions to obtain sufficient seeds for this study. Homozygous insertion mutants were identified by investigating ten progenies from each of the knockout mutants by conducting polymerase chain termination reaction (PCR) (describe in a separate section below). At least two homozygous knockout mutants were investigated for each candidate gene. The knockout mutants and wild-type Col-0 ecotype were phenotyped under control and prolonged cold-stress conditions.

### Genome-wide association studies

The GWAS was conducted for the average trait value (changes in the rosette area under extended cold stress) of the accessions under each biological replicate. The GWAS was performed in the easyGWAS web interface (Grimm et al., 2017) using two popular genome-wide association tools, the linear regression (LR) model or Efficient Mixed-Model Association eXpedited (EMMAX). The EMMAX model (Kang et al., 2010) allows for correcting false correlations due to population structure, and the LR model finds genetic variants linked with continuous traits in GWAS (Wang et al., 2018). Quantile-quantile plots (QQ plots) providing information regarding the genomic control factor on the easyGWAS platform were utilized to evaluate the performance of the EMMAX or LR model in controlling for the *p*-value inflation caused by population structure. The 1001 Genomes collection (Alonso-Blanco et al., 2016) was utilized in the easyGWAS platform (Grimm et al., 2017), which gives an exceptional opportunity to comprehend how genetic variation translates into phenotypic variation and to explore the numerous ways in which plants respond to environmental challenges. A total of 6,973,565 SNPs with a minor allele frequency >0.05 were used. Major SNP linkage disequilibrium plots were also generated on the easyGWAS platform (Grimm et al., 2017). When a strongly linked SNP was found to co-localize with the exon/intron or promoter (2-kb upstream) regions of a gene, the gene is considered as a candidate chilling tolerance gene.

### Identification of homozygous T-DNA and transposon insertion mutants

We studied at least two independent T-DNA insertion mutants for each candidate gene to validate the chilling-tolerance function of the putative genes identified by GWAS. The information about primer sequences, insertion locations, and the estimated T-DNA confirmation product size was obtained from the T-DNA Primer Design site (http://signal.salk.edu/tdnaprimers.2.html). The homozygous plants for any T-DNA insert from individual segregants were identified essentially by a two-step PCR genotyping assay (O’Malley et al., 2015).

A gene/genome-specific primer (GSP: LP, RP - Left, right genomic primer) pair spanning the predicted T-DNA insertion site was used for the first PCR reaction to detect the presence of a wild-type copy (WT copy) of the gene in the wild type or heterozygous individuals. However, no band was amplified for a homozygous plant because both copies of the gene contain the T-DNA insert, whose large size precludes PCR amplification. The lack of a wild-type gene-specific PCR provided strong evidence that the line is homozygous for the insert. The second PCR reaction was used to validate the homozygosity for a T-DNA insert in the gene. In Col-0, we failed to amplify the T-DNA inserted genomic region in the second PCR, while a T-DNA and target insertion site-specific PCR product was amplified in the heterozygous and homozygous T-DNA insertion mutants. The homozygous lines showed a lack of the gene-specific and presence of T-DNA insertion site-specific PCR amplification. The heterozygotes showed amplification of both types of PCR products, i.e., gene-specific and T-DNA insertion site-specific. The T-DNA insertion site was selectively amplified using a combination of a left border primer (LB - the left T-DNA border primer) and the correctly oriented GSP primer (LP or RP) specific to the target insertion site.

The only transposon insertion CS26712 line carrying a unique insertion of an enhancer trap (ET) transposable *Ds* element disrupting the *AT2G18260* gene function was screened using primers specific to the flanking sequences of the insertion site. *AT2G18260*-specific primers together with the insertion site specific primers were used to identify the homozygous mutants as in the characterization of T-DNA insertion mutants.

### BLAST search and MapMan analyses

Blast2GO was used to determine the function and localization of the candidate genes. Blast2GO is a widely used annotation platform that uses homology searches to associate sequences with Gene Ontology (GO) terms and other functional annotations (Conesa et al., 2005; BioBam Bioinformatics, 2019; Sahoo et al., 2022). Blast2GO generated Gene Ontology annotations based on (a) Biological process, (b) Molecular Function, and (c) Cellular Component.

To display cold stress-responsive genes onto pathways, the MapMan (Usadel et al., 2009) was used to analyze the 16 chilling tolerance genes (**Table 1**) and nine strong candidate chilling tolerance genes (**Table 2**) that are either induced or suppressed in response to cold stress.

**Table 1:** The 16 identified Arabidopsis genes involved in the expression of chilling tolerance.

Sl. No.	Gene ID	Protein ID	^1^Expression	Mutant	^2^Phenotype	^3^Algorithm
1	AT4G14400	Accelerated cell death 6 (ACD6)	0.06	SALK_059132	1.73	Linear Regression
				SALK_116079	1.46	
2	AT5G02360	Domain C1 containing protein	0.09	SALK_004531	1.75	Linear Regression
				SALK_004524	2.46	
3	AT2G18260	AtSYP112	0.14	SALK_037621	2.47	EMMAX & Linear Regression
				CS26712	2.21	
				CS868715	1.87	
4	AT1G61310	NB-ARC LRR	0.43	SALK_125189	2.66	EMMAX & Linear Regression
				SALK_029306	1.56	
5	AT2G19110	Heavy metal ATPase 4	0.5	SALK_042898	2.1	EMMAX
				SALK_093482	3.65	
6	AT5G41750	TIR-NB-LRR	0.53	SALK_066101	1.34	EMMAX & Linear Regression
				SALK_085020	1.64	
7	AT5G23420	High-mobility group box 6	0.62	SALK_138632	2.12	Linear Regression
				SALK_044693	1.56	
8	AT5G52460	F-box leucine-rich repeat protein	0.71	SALK_013776	2.77	EMMAX
				SALK_031583	0.39	
9	AT3G61600	LRB2; POZ/BTB containing G-protein 1	0.76	SALK_100118	2.36	Linear Regression
				SALK_128387	2.78	
10	AT3G24900	Receptor-like protein 39	1.31	CS868997	0.74	EMMAX
				SALK_126504	0.6	
11	AT1G31870	Bud site-selection protein 13	1.52	SALK_018219	1.77	EMMAX & Linear Regression
				SALK_096851	1.58	
12	AT2G19060	SGNH hydrolase-type esterase	1.68	SALK_061864	2.47	EMMAX & Linear Regression
				SALK_117794	1.72	
				SALK_115819	1.86	
13	AT2G04300	Leucine-rich repeat protein kinase	4.37	SALK_003316	2.3	EMMAX & Linear Regression
				SALK_003328	1.85	
14	AT4G12040	Stress-associated protein 7	5.97	SALK_071408	-1.03	Linear Regression
				SALK_071504	0.58	
15	AT4G12000	SNARE associated Golgi protein	10.37	SALK_021373	0.51	EMMAX
				SALK_204172	0.67	
16	AT2G31360	16:0 delta 9 desaturase 2	26.04	SALK_079963	0.05	EMMAX
				SALK_016783	-0.49	

^1^The expression ratio indicates the proportion between the average transcript levels under low versus normal growing temperature conditions. The averages were calculated from the data presented in **Figure S3**.

^2^Proportion of pixilated growth data of mutant versus wild-type Col-0 ecotype under cold stress at 4 °C for 30 days. Data are taken from **Figures 2**, **3**, and **Figure S5**.

^3^EMMAX and Linear Regression models representing possible population structures were used in GWAS. Both models were used in multiple GWAS. Models that identified the genes are shown.

**Table 2:** The nine strong candidate genes involved in adapting Arabidopsis to cold stress.

Sl. No.	Gene ID	Protein ID	Mutant	^1^Mutant Phenotype	^2^Expression Level	^3^Algorithm
1	AT3G61480	Quinoprotein amine dehydrogenase	*SALK_017426	1.49	0.84	Linear
			SALK_099827	0.96	0.84	
2	AT1G31640	Agamous-like 92	*SALK_030847C	1.79	0.85	EMMAX
			SALK_035114C	1.17	0.85	
3	AT2G27120	DNA polymerase epsilon catalytic subunit	*SALK_025607	2.52	0.92	Linear
			SALK_056503	0.81	0.92	
4	AT3G53520	UDP-glucuronic acid decarboxylase 1	*SALK_068865	1.36	1.07	EMMAX & Linear
			SALK_152673	1.25	1.07	
5	AT2G39840	Type 1 phosphoprotein Ser/Thr phosphatase	SALK_090980	0.97	1.34	EMMAX & Linear
			SALK_090981	1.22	1.34	
			*CS375515	-0.73	1.34	
6	AT4G12350	AtMYBb42	*SALK_003422	1.28	1.37	EMMAX
			SALK_032016	0.86	1.37	
7	AT5G54960	Pyruvate decarboxylase-2	*SALK_053107	0.48	2.43	EMMAX
8	AT3G43148	Myosin heavy chain-like protein	*SALK_151592	0.55	n/a	EMMAX
			SALK_076725	0.96	n/a	
9	AT5G39500	Endoplasmic reticulum morphology 1	*SALK_020371	0.57	n/a	EMMAX
			SALK_091078	1.21	n/a	

^1^The expression ratio indicates the proportion between the average transcript levels under low versus normal growing temperature conditions. The averages were calculated from the data presented in **Table S5**.

^2^Proportion of pixilated growth data of mutant versus wild-type Col-0 ecotype under cold stress at 4°C for 30 days. Data are taken from **Figures 2**, **3**, and **Figure S5**.

^3^EMMAX and Linear Regression models representing possible population structures were used in GWAS. Both models were used in multiple GWAS. Models that identified the genes are shown. n/a, data not available.

### Statistical analysis

Using package R program version 1.6.1 (Ihaka and Gentleman, 1996), the Student’s t-test was performed to determine the significant differences in the comparative growth rates of mutants to Col-0 under control and prolonged cold-stress conditions; whereas, one-way ANOVA (Analysis of Variance) was performed to determine growth rate differences between the ecotypes.

## Results

### A high-throughput digital image-based two-dimensional (2D) phenotyping platform for rosette leaves of Arabidopsis seedlings

**Figure 1:**
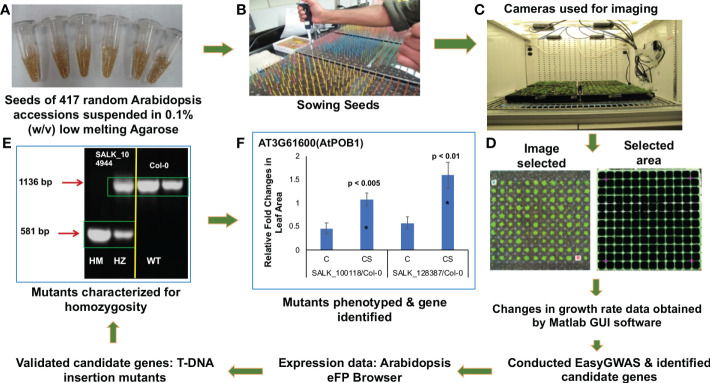
Schematic representation of the steps in identifying genes involved in cold stress using a high-throughput phenotyping platform. After suspending in low melting agarose **(A)**, seeds were stratified, and sown **(B)**, and grown in the Arabidopsis growth chambers fitted with digital cameras **(C)**. Two-dimensional images of the rosette leaves of individual genotypes were captured and analyzed using Matlab GUI software (**Method S2**) **(D)**. The digital images were converted to pixel data for easyGWAS to identify putative cold-stress-related genes. The candidate cold-stress-related genes were validated by studying knockout mutants. Homozygous knockout mutants were identified **(E)**. SALK_104944, knockout T-DNA insertion mutant for *AT1G68320;* HM, homozygous line; HZ, heterozygous line; WT (Col-0 ecotype), wild-type with no insertion. Knockout mutants were phenotyped to determine if any of the 33 putative cold-stress-related genes play a role in chilling tolerance **(F)**.

We resuspended the surface sterilized seeds in 0.1% (w/v) NuSieve Agarose (see Materials and Methods for details) (**Figure 1A**). The accessions were randomized within each of the three blocks in a randomized block design. The day of sowing the seeds was counted as “Day 0”. The plants were grown in AR-22L Arabidopsis chambers (Percival, IA, USA) (**Figure 1C**). Four days after germination, seedlings were either thinned for a group of ten plants or transplanted to obtain one plant per cell. Plants were grown under 16 h day with light intensity 100 *μ*mol m^−2^ s^−1^, 22°C temperature, 50% relative humidity (RH), and 8 h night with 18°C temperature and 60% RH. The plants were watered once a week. On Day 7, in the “cold-stress” group, the temperature was reduced to 4°C as the day and night temperature for 30 days, while plants in the “control group” continued to grow with no changes in growing conditions for seven additional days.

In the mutant studies, each tray contained the cold-tolerant accession PYL-6, the cold-sensitive accession Stepn-2, and Columbia-0 (Col-0) and overlapping mutant lines to facilitate comparisons across an experimental group or between independent experiments to assure reproducibility. For each genotype, three experiments were conducted. In each experiment, ten seedlings were planted in each of 20 random cells in the “group of plants study.” Thus, each phenotypic observation represented datum collected from a group of ten plants of a cell; and for each genotype, data were collected from 60 cells (n = 60 from three experiments).

Two-dimensional (2D) images of the rosette leaves of a single or group of plants were captured by CropScore cameras (Computomics GmbH, Tübingen, Germany) during the day (**Figure 1D**) and stored in the CropScore server (http://www.cropscore.com/en/home.html) for further analysis.

### Image analyses of the 2D images using the Matlab GUI software

A user-friendly software program Matlab GUI was written in Matlab to (i) capture and store a large dataset of high-resolution 2-dimensional (2D) digital images of aerial views of Arabidopsis rosette leaves in growth chambers over a period of time until leaves of the adjacent single or clusters of plants start to overlap; (ii) automatically crop, register and segment high-resolution images into sub-images corresponding to individual accessions based on a sequence of computer vision techniques (**Method S1**); (iii) extract important cues like rosette color and total rosette area from each sub-image, and store these cues in spreadsheet format for downstream statistical analysis (**Method S2**). Each of these stages is automatic, with an option included for manual intervention.

Our image processing workflow (**Methods S1**-**S2**) executes the following six steps: (1) The Arabidopsis growth chambers are equipped with a network of point-and-shoot four cameras obtained from CropScore Inc (Computomics GmbH, Tübingen, Germany). Each Plug Tray-288 is divided into two halves, and each carrying 144 wells (12 x 12 wells) that are photographed by a single camera. Images are captured every 12 hours during the light period and transferred to a central server *via* an Ethernet connection (**Figure 1C**). This step can be automated. (2) The images are cropped by detecting the tray boundaries if the camera is misoriented. This step can be automated. (3) For each image, the boundaries between adjacent cells in the grid are automatically detected using a two-step process (**Figure 1D**): (i) each image is passed through a color filter tuned to the tray color, and (ii) edge boundaries, which are linear features in the filtered image, are estimated using a Hough transform. This step can be automated. (4) The 2D plane of the tray is estimated by calculating the geometric intersections of the cell boundaries. A perspective correction is applied to the original unfiltered image to register it to an orthographic view with respect to this plane. This step can be automated. (5) The registered image is now segmented into sub-images of different cells by simple cropping. This step can be automatic. (6) Cell images are passed through a second color filter tuned to the rosette leaves of healthy Arabidopsis. Rosette areas are then estimated by aggregating adjacent pixels, which have high responses to this color filter. This step can be made automatic. Steps 2 and 3 are the most challenging, and sometimes the automatic cropping and registration can fail if the tray boundaries are not detected correctly. If this is the case, the workflow signals the user to manually crop the image using a graphical user interface (GUI).

**Figure 2:**
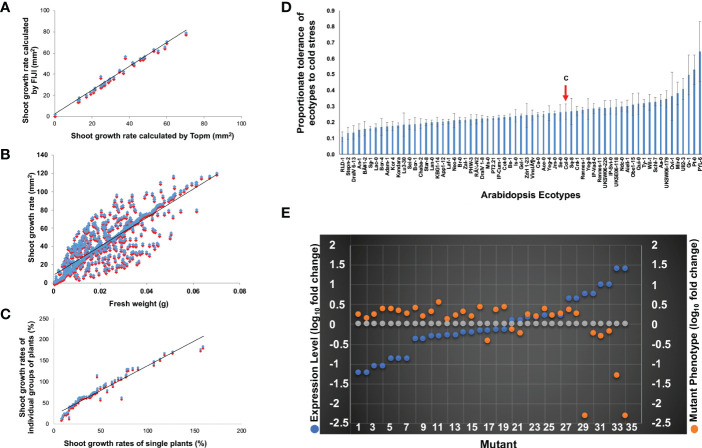
Phenotypic analyses of the chilling tolerance trait in Arabidopsis. **(A)** Scatterplots demonstrating the relationships between the shoot surface area calculated by FIJI (blue) and Top.m (red) programs. The fitted line displays statistically significant correlations (*r* = 0.99). **(B)** Relationships between the digital shoot surface area (sq. mm) calculated by Top.m (blue spot) and fresh weight (g) of shoots. The scatterplot showed a positive association (*r* = 0.83) between the digital shoot surface area (sq. mm) calculated by Top.m (blue) and fresh weight (g) of shoots (red spot) among the 417 ecotypes. **(C)** Scatterplot demonstrating the positive association (*r* = 0.96) between the digital shoot surface area (%) from a group of plants (blue) with that of the corresponding single plant (red) of 76 randomly selected ecotypes. Growth Rate (%) in a-c was calculated as follows: [(Final Growth – Initial Growth)*100]/Initial Growth. **(D)** Tolerances of 417 Arabidopsis ecotypes to continuous cold stress. The growth rate of each ecotype (%) is calculated as = Growth on the 30^th^ day of Cold Treatment X 100/Growth on the 0^th^ Day of Cold Treatment. The proportionate tolerance of each ecotype is calculated as the growth rate of each ecotype X 100/the summation of the growth rate of 417 ecotypes (detailed information is in **Figure S2**). The red arrow shows the proportionate growth of the ecotype Col-0. **(E)** Relationship of steady-state expression levels and mutant phenotypes of 16 chilling tolerance genes. All but one knockout mutant of nine genes with reduced steady-state transcript levels under cold stress showed enhanced growth rates compared to wild-type ecotype Col-0 in response to prolonged low-temperature exposure (orange dots). Knockout mutants of four genes with enhanced steady-state transcript levels during cold stress showed reduced growth rates compared to wild-type ecotype Col-0 in response to prolonged cold stress. Grey dots showed log_10_ of 1 for transcript levels of genes or growth levels of mutants with no change at 4°C compared to wild-type Col-0. The data are from **Table 1**. Expression levels of individual genes (blue dots) at 24 h following exposure to cold stress (**Figure S3**) were used to plot the phenotypes of mutants identified for that gene.

The utility of our software for two-dimensional (2D) Arabidopsis rosette leaf image analyses was evaluated through the analysis of a set of random accessions (**Figure 2A**) as follows. The images of 144 accessions were processed and analyzed by both FIJI (Abràmoff et al., 2004; Schindelin et al., 2012) and our Matlab software GUI (**Method S1**-S2). The 2D rosette leaf areas calculated by the two software for 144 accessions showed a statistically significant positive association (*r* = 0.99; *p <*0.01; **Figure 2A**). Our Matlab GUI is a user-friendly software; therefore, we used this software to analyze the images. This approach’s key benefit is that it minimizes the amount of manual intervention and reduces data processing times by a factor of at least 20 compared to that for the existing commercially available phenotyping software solutions, such as the FIJI software used in this investigation for evaluating the performance of Matlab GUI (D.K. Sahoo, C. Hegde and M.K. Bhattacharyya, unpublished).

We investigated if the 2D aerial images of Arabidopsis rosette leaves correctly predict the leaf growth of individual accessions. The 2D rosette leaf area of 417 accessions was determined using our software. The fresh weights (g) of rosette leaves of the same 417 accessions were also measured. The correlation coefficient between the 2D leaf area and fresh leaf weight (g) among the 417 accessions was *r* = 0.83 (*p*<0.01; **Figure 2B**), suggesting the suitability of the phenotyping system for the Arabidopsis seedlings.

The growing of single Arabidopsis plants is labor-intensive. Therefore, we investigated if the 2D leaf area of ~15 plants can predict the 2D leaf area of a single plant. The association between the 2D image-based growth of single plants with that of groups of ~15 plants among 76 accessions was found to be highly significant (r = 0.96; *p <*0.01; **Figure 2C**), suggesting that investigation of groups of plants instead of single plants should provide a reliable fresh weight estimate for an accession.

### Responses of Arabidopsis ecotypes to prolonged cold stress

Using our 2D aerial rosette leaf phenotypic system, we investigated the responses of 417 Arabidopsis accessions, genomes of which have been resequenced (Alonso-Blanco et al., 2016), to prolonged cold-stress (4°C). The 417 accessions include accessions studied previously for responses to non-freezing (Barah et al., 2013) and freezing cold stresses (Zhen and Ungerer, 2008; Xie et al., 2019). We collected the estimated 2D aerial rosette leaf areas of the selected 417 accessions under either 22° C (C) or prolonged cold stress at 4 °C (CS) in square mm (**Table S2**; **Figure S2**). A 10-fold difference in the 2D aerial leaf area was observed between the most chilling-tolerant accession, PYL-6, and the most chilling-sensitive accession, Stepn-2 (**Figure 2D**; **Figure S2**). The broad-sense heritability (*h^2^*) for the aerial rosette leaf phenotype was 84%, suggesting that the phenotype is less influenced by environment and therefore reliable.

### 33 A genome-wide association study revealed putative chilling tolerance genes

The 417 lines considered for this study were resequenced previously, and single nucleotide polymorphisms (SNPs) for the entire genome were predicted by comparing its genome sequence with the reference genome sequence of Columbia-0 (Col-0) (Grimm et al., 2017). We conducted a GWAS of the phenotypic variation of the 417 accessions for leaf responses to prolonged cold stress with the SNPs distributed across the entire genome (Grimm et al., 2017). Pixel data of the natural variants were log-transformed to facilitate reliable parametric tests. The association of the phenotypic data with SNP data was tested using both (i) linear regression (LR) or (ii) an efficient mixed-model association expedited (EMMAX) model (Grimm et al., 2017). An arbitrary cutoff *p-*value of –log10 ≥ 4.5 identified 33 genes (Kaler and Purcell, 2019). The GWAS was conducted for each of the 17 independent experiments (CS; Cold Stress) and the mean of all data from the 17 experiments (**Table S3**). Quantile-quantile (QQ) plots (**Figure S1B**) suggested that the data are normally distributed. The frequency distribution of the 417 accessions for proportionate chilling tolerance also exhibited a normal distribution (**Figure S1C**). Of the 33 genes, 15 were identified when the EMMAX model was used, and seven when the LR model was used. Eleven genes were detected in both LR and EMMAX models (**Table S4**).

### Expression patterns of the 33 candidate chilling tolerance genes

A large number of genes are transcriptionally regulated in response to cold stress (Winter et al., 2007). Therefore, we investigated the 33 candidate chilling tolerance genes for their expression levels from the microarray data of leaf samples from Arabidopsis plants exposed to 4 ^0^C for 24 h. We downloaded the expression data from the “Electronic Fluorescent Pictograph (eFP)” database (Winter et al., 2007). To our surprise, 32 of the 33 genes are regulated at the transcriptional level to some extent in response to cold stress (**Figure S3**; **Table S5**). This observation suggests that at least some of the 33 genes could be involved in adapting Arabidopsis to prolonged cold stress.

### Analyses of insertion mutants identified 16 genes involved in chilling tolerance

We investigated 64 homozygous T-DNA insertion- and one transposon-induced mutants for the 33 candidate chilling tolerance genes. We were able to identify two or more homozygous insertion mutants for 29 genes, with one mutant each for the remaining four genes (**Figure 1E, F**; **Figure S4**; **Table 1**; **Table S6**, **S7**). At least two mutants for each of the 16 of 29 genes showed significant differences in rosette leaf growth from the wild-type Col-0 following exposure to 4°C for 30 days (**Table 1**). We termed these 16 genes as chilling tolerance genes. We were able to observe altered mutant phenotypes in only one mutant for each of the nine genes (**Table 2**; **Table S7**). These nine genes may govern subtle chilling tolerance phenotypes. It will require additional studies, including complementation analysis, to understand their function in chilling tolerance. We consider these nine genes as strong candidate genes for chilling tolerance. Both tested mutants failed to show any altered mutant phenotypes for the remaining four of the 29 genes (**Table S7**).

We investigated if there was any relationship between levels of transcripts and responses of the knockout mutants of the identified 16 chilling tolerance genes to prolonged cold stress. We hypothesized that knockout mutants of the genes with reduced transcript levels under cold stress would have enhanced cold tolerance, while mutants of the genes induced during cold stress would show enhanced sensitivity to cold stress. Mean transcript levels for each of the 16 genes were calculated from six data points (**Table 1**; **Figure S3**). Each of the 16 genes showed significant up- or down-regulation for at least one of the six data points (Winter et al., 2007) (**Figure S3**). We did observe a clear relationship for thirteen of the 16 genes as expected; knockout mutants for nine genes with reduced transcript levels under cold stress showed enhanced chilling tolerance, while mutants of four genes with increased transcript levels under cold stress exhibited increased cold sensitivity (**Figure 2E**; **Table 1**).

### Functions of the identified 16 chilling tolerance genes

**Figure 3:**
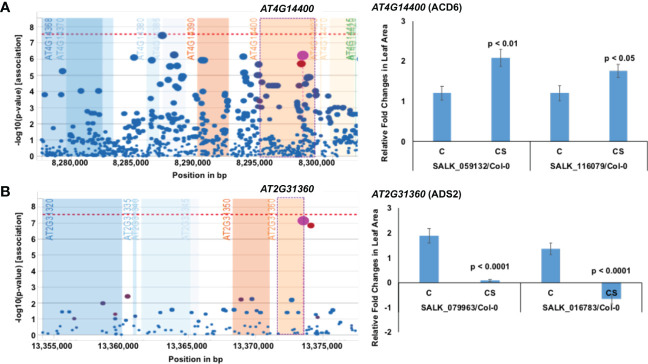
Previously identified two proteins that contribute either negatively or positively to chilling tolerance. **(A)** *AT4G14400* encoding Accelerated cell death 6 (ACD6) protein negative regulates chilling tolerance. **(B)** *AT2G31360* encoding acyl-lipid desaturase 2 (ADS2) positively regulates chilling tolerance. On the left, output plot of *p*-values (−log base 10) in a 5-kb window for association of SNPs with phenotypic variation, obtained from easyGWAS, is presented. On the right, rosette leaf growth rates of mutants with respect to Col-0 are presented. The relative rosette leaf growth rate in the mutant compared to wild-type Col-0 is significantly different in control **(C)** and cold stress (CS) (*p* < Bonferroni adjusted α) conditions. Knockout mutation in the *ADS2* gene resulted in yellowing and death of plants resulting in a negative growth rate in one T-DNA insertion mutant at 4°C and as compared to that at 22°C. C, Comparative growth rate of the mutant relative to wild-type Col-0 in control condition; CS, Comparative growth rate of the mutant relative to wild-type Col-0 in cold stress.

Interestingly only two of the 16 genes identified have previously been reported as chilling tolerance genes: *AT4G14400*, which conditions accelerated cell death 6 (ACD6), and *At2g31360*, which encodes an acyl-lipid desaturase 2 (ADS2). *ACD6* (*AT4G14400*) is downregulated by cold stress, and its loss of function knockout mutants showed enhanced chilling tolerance to cold stress (**Figure 3**; **Table 1**; **Figure S3A**). This gene has been shown to be involved in chilling and freezing tolerance (Chen and Thelen, 2013). A gain of function *acd6* mutant shows an increased accumulation of salicylic acid level and exhibits freezing sensitivity (Lu et al., 2009; Miura and Ohta, 2010; Chen and Thelen, 2013) along with enhanced resistance against both bacterial and oomycete pathogens (Todesco et al., 2010).

*ADS2* (*At2g31360*) is upregulated by cold stress, and the *ads2* mutant was shown to display a dwarf and sterile phenotype in response to cold stress at 6°C and to show increased sensitivity to freezing temperature (Chen and Thelen, 2013). Here we also observed that the mutants of the cold-induced *ADS2* gene showed increased sensitivity to cold stress (**Figure 3B**; **Table 1**; **Figure S3B**). In *ads2* mutant plants, the membrane lipid composition is altered compared to the wild-type plants. Reduced levels of 16:1, 16:2, 16:3, and 18:3 lipids and higher levels of 16:0 and 18:0 fatty acids were detected in the *ads2* mutant compared to the wild-type plants. The paralogous *acyl-lipid desaturase 1* and *2* (*ADS1* and *ADS2*) genes are induced in response to cold stress to facilitate cold acclimation and chilling/freezing tolerance, respectively (Chen and Thelen, 2013; Chen and Thelen, 2016). *ADS1* encodes a soluble Δ9-desaturase that is found primarily in the chloroplast and catalyzes the desaturation of stearic acid (18:1) of monogalactosyl diacylglycerol (MGDG) (Barrero-Sicilia et al., 2017; Berestovoy et al., 2020); while *ADS2* encodes a 16:0 desaturase for the synthesis of MGDG and phosphatidylglycerol (Chen and Thelen, 2013). Both genes affect chloroplast membrane desaturation and have been shown to be essential for the cold adaptation response in Arabidopsis (Barrero-Sicilia et al., 2017). The re-identification of *ACD6* and *ADS2* cold tolerance genes validates our approach of identifying chilling tolerance genes using a novel phenotyping system for Arabidopsis seedlings.

**Figure 4:**
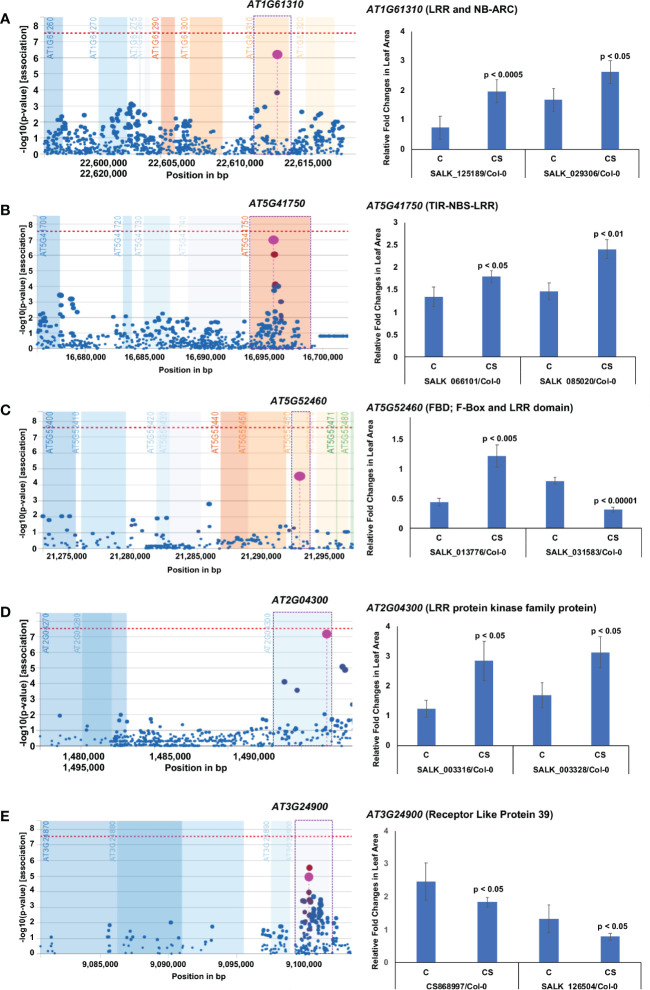
Five leucine-rich repeat domain-containing proteins contribute towards chilling tolerance. **(A–E)** Each of the five proteins contains LRR domain. On the left, output plot of *p*-values (−log base 10) in a 5-kb window for the association of SNPs with phenotypic variation, obtained from easyGWAS, is presented. On the right, rosette leaf growth rates of mutants with respect to Col-0 are presented. The relative rosette leaf growth rate in the mutant compared to wild-type Col-0 is significantly different in control **(C)** and cold stress (CS) (*p* < Bonferroni adjusted α) conditions. C, Comparative growth rate of the mutant relative to wild-type Col-0 in control condition; CS, Comparative growth rate of the mutant relative to wild-type Col-0 in cold stress.

Five of the 14 novel chilling tolerance genes identified in this study contain LRR domains with unknown functions (**Figure 4**; **Table 1**). *AT1G61310* encodes an LRR and NB-ARC domains-containing disease resistance-like protein, while *AT5G41750* encodes a TIR-NBS-LRR-type disease resistance-like protein. *AT5G52460* encodes an F-box leucine-rich repeat protein, annotated as embryo sac development arrest 41 (EDA41). The transcript levels of all three genes were suppressed by cold stress (**Figure S3C-E**). All knockout mutants except one for these three genes showed enhanced chilling tolerance compared to the wild-type Col-0 ecotype; one T-DNA insertion mutant, SALK_031583, for the *AT5G52460* gene showed cold sensitivity (**Figure 4A-C**; **Table 1**). In the SALK_031583 mutant, T-DNA was inserted in the promoter region, which might have enhanced the transcription of the gene leading to increased cold sensitivity. *AT2G04300* encodes an LRR protein kinase, and *AT3G24900* encodes the receptor-like protein 39 (AtRLP39) containing an LRR domain. The transcript levels of these two genes are induced during cold stress (**Figure 4D, E**; **Table 1**; **Figure S3F-G**). Surprisingly, although the *AT2G04300* gene is highly induced, the knockout mutants showed increased growth instead of reduced growth compared to the control wild-type Col-0 ecotype under prolonged cold stress (**Figure 4D**; **Table 1**; **Figure S3F**). The knockout mutants of the cold stress-induced gene *AT3G24900* exhibited reduced growth compared to that in the wild-type Col-0 ecotype under prolonged cold stress (**Figure 4E**; **Table 1**; **Figure S3G**).

In addition to assigning the function of chilling tolerance to five unique LRR domain-containing proteins (*AT1G61310* encoding NB-ARC LRR, *AT5G41750* encoding TIR-NB-LRR, *AT5G52460* encoding F-box leucine-rich repeat protein, *AT2G04300* for Leucine-rich repeat protein kinase and *AT3G24900* encoding Receptor-like protein 39), we ascribed the function to nine additional genes. They are: (i) *AT1G31870* encoding the bud site-selection protein 13 (AtBUD13), which is involved in pre-mRNA splicing and embryo development (Xiong et al., 2019); (ii) *At5g02360* encoding DC1 domain-containing protein, (iii) *AT2G18260* encoding syntaxin of plants 112, (iv) *AT2G19110* encoding heavy metal ATPase 4, (v) *AT5G23420* encoding high-mobility group box (HMGB) 6, (vi) *AT3G61600* encoding LRB2; POZ/BTB containing G-protein 1, (vii) *AT2G19060* encoding SGNH hydrolase-type esterase, (viii) *AT4G12040* encoding stress-associated protein 7, and (ix) *AT4G12000* encoding SNARE associated Golgi protein (**Table 1**).

*AT1G31870* encodes the bud site-selection protein 13 (AtBUD13) (**Table 1**; **Figure S3H**; **Figure S5A**), which is involved in pre-mRNA splicing of 52 genes, of which 22 are involved in nucleic acid metabolism and embryo development (Xiong et al., 2019). Cold stress alters the expression and splicing of serine/arginine-rich (SR) genes that encode splicing factor proteins required for constitutive and alternative splicing (Palusa et al., 2007; Leviatan et al., 2013). *AtBUD13* could be a regulatory gene controlling cold-stress-related genes for cold adaptation through splicing.

Transcription of *AT2G18260* gene encoding syntaxin protein ATSYP112, also suppressed by cold stress loss of function mutants are chilling tolerant; therefore, we consider that this protein negatively contributes towards chilling tolerance in Arabidopsis (**Table 1**; **Figure S3I**; **Figure S5B**).

*AT2G19060* encodes an SGNH hydrolase-type esterase (**Table 1**; **Figure S3J**; **Figure S5C**). The GDSL esterases/lipases are mainly involved in regulating plant development, morphogenesis, synthesis of secondary metabolites, and defense response (Hong et al., 2008; Kwon et al., 2009; Chepyshko et al., 2012). The GDSL family is further classified as SGNH hydrolase because of the presence of the strictly conserved residues Ser-Gly-Asn-His in the conserved blocks I, II, III, and V (Chepyshko et al., 2012). The role of GDSL family esterase in cold adaptation was reported in *Photobacterium* sp. strain J15 (Shakiba et al., 2016). Here, we reported a SGNH hydrolase-type esterase as a negative regulator during the cold stress response.

*AT2G19110* encodes the Arabidopsis heavy metal ATPase 4 (AtHMA4) with similarity to Zn ATPase (Meyer et al., 2016; Lekeux et al., 2018). Transcription of this gene is downregulated by cold stress, and knockout mutants show enhanced cold tolerance suggesting a negative role of *AtHMA4* in cold tolerance (**Table 1**; **Figure S3K**; **Figure S5D**). *ATHMA4* is involved in the hyperaccumulation of Zn/Cd (Lekeux et al., 2019). Presumably, metal ion accumulation may be detrimental during cold stress. A study in the halophyte four-wing saltbush (*Atriplex canescens*) revealed a heavy metal-associated protein, AcHMA1, whose expression was strongly downregulated under NaCl and cold stress (Sun et al., 2014). In Arabidopsis, such a mechanism might be mediated by *ATHMA4*, downregulated under cold stress.

*AT3G61600* encodes the LIGHT-RESPONSE BTB2 (LRB2) protein, which, together with LRB1, negatively regulates photomorphogenesis (Christians et al., 2012). This gene is involved in protein ubiquitylation through interacting with CULLIN 3 proteins (Gingerich et al., 2005; Christians et al., 2012; Hu et al., 2014). In winter-annual accessions of *A. thaliana*, cold stress exposure or vernalization is needed to commence flowering *via* FRIGIDA (FRI). FRI acts as a scaffold protein to recruit numerous chromatin modifiers that epigenetically modify flowering genes and regulate blooming *via* proteasome-mediated degradation of FRI. During vernalization, FRI directly interacts with the BTB proteins LRB1/2, as well as the CULLIN3A (CUL3A) ubiquitin-E3 ligase *in vitro* and *in vivo* leading to proteasomal degradation of FRI (Christians et al., 2012; Hu et al., 2014). Long-term cold stress accelerates the degradation of FRI and blooming by decreasing FLC transcription, a mechanism dependent on CUL3A and associated with long non-coding RNA and chromatin remodeling (Hu et al., 2014). Here we have shown that the transcription of *LRB2* is suppressed by cold stress and knockout mutants showed enhanced chilling tolerance suggesting its negative function for chilling tolerance (**Table 1**; **Figure S3L**; **Figure S5E**).

The *AT4G12000* gene encodes a member of the soluble N-ethylmaleimide-sensitive factor adaptor receptor (SNARE)-associated Golgi protein family (Xu et al., 2019). Arabidopsis, 53 genes have been annotated to encode SNARE molecules (Sanderfoot et al., 2000). SNARE proteins in plants are involved in various physiological processes, including abscisic acid-related signaling and osmotic stress tolerance (Uemura et al., 2004; Bassham and Blatt, 2008), and soluble N-ethylmaleimide-sensitive factor adaptor protein (SNAP) genes are induced at low temperature (Bao et al., 2008). The expression of *OsSNAP32* was dramatically increased in rice seedlings treated with H_2_O_2_, PEG6000, and low temperature or after inoculation with the rice blast pathogen *Megnaporthe oryzae*. A gene family encoding SNAP25-type proteins is induced in rice following exposure to biotic and abiotic stresses (Bao et al., 2008). *AT4G12000* encoding a SNARE-associated Golgi protein identified in the present study is also induced under cold stress, and loss of function mutants for this gene displayed chilling-sensitive phenotypes suggesting a positive function for this gene in chilling tolerance (**Table 1**; **Figure S3M**; **Figure S5F**).

*AT4G12040* encodes an A20/AN1-like zinc finger family protein, stress-associated protein 7 (ATSAP7) (Vij and Tyagi, 2006). A20/ANI zinc-finger domain-containing SAPs are involved in abiotic stress (Mukhopadhyay et al., 2004). Another SAP, AtSAP10, involves various abiotic stresses such as heavy metals and metalloids, high and low temperatures, and treatment with ABA (Dixit and Dhankher, 2011). *AtSAP12* is induced following cold treatment (Ströher et al., 2009). Expression of both *OsiSAP1* and *OsiSAP8* is induced in rice in response to a variety of environmental stresses, including cold, drought, heavy metals, wounding, and submergence (Mukhopadhyay et al., 2004; Kanneganti and Gupta, 2008) and overexpression of *OsiSAP8* provides rice with strong tolerance to cold, salt, and drought (Mukhopadhyay et al., 2004; Kanneganti and Gupta, 2008). In tobacco, overexpression of *ZFP177*, a rice zinc-finger *A20/AN1* gene, enhanced tolerance to high and low temperatures (Huang et al., 2008). Similarly, overexpression of AlSAP, a stress-associated protein from a halophyte grass *Aeluropus littoralis*, in tobacco provides increased tolerance to cold, heat, salt, and drought stresses (ben Saad et al., 2010). Expression of *ATSAP7* (*AT4G12040*) is induced by cold stress, and both T-DNA insertion knockout mutants identified for this gene exhibited reduced growth rate under prolonged cold stress as compared to that in the wild-type Col-0 ecotype, a positive chilling tolerance function as observed for other Arabidopsis and rice SAP proteins containing A20/AN1-like zinc finger domains (**Table 1**; **Figure 3N**; **Figure S5G**).

*At5g02360* encodes a cysteine/histidine-rich divergent C1 (DC1) domain-containing novel protein (Allen et al., 2004); **Figure S5H**). The transcription of this gene is suppressed in response to cold stress (**Figure S3O**). A cysteine/histidine-rich DC1 protein has been shown to positively regulate cell death and plant defenses in pepper (Hwang et al., 2014). Here we have shown that knockout mutants for *At5g02360* are highly tolerant to cold stress compared to the wild-type control, suggesting a possible negative function for this gene (**Table 1**; **Figure 3O**; **Figure S5H**).

*AT5G23420* (**Table 1**; **Figure S3P**; **Figure S5I**) encodes a high-mobility group box 6 (HMGB6) protein (Grasser et al., 2004; Kwak et al., 2007; Pedersen and Grasser, 2010). HMGB nuclear proteins are involved in various cellular processes, including replication, transcription, and nucleosome assembly. The Arabidopsis genome contains eight genes encoding HMGB proteins (Grasser et al., 2004; Kwak et al., 2007). Cold treatment increases the expression of *HMGB2, HMGB3*, and *HMGB4*, whereas the transcript levels of *HMGB1* and *HMGB5* are not noticeably affected by cold stress (Kwak et al., 2007). The expression of *AT5G23420* is suppressed by cold stress, and both T-DNA insertion knockout mutants for *AT5G23420* exhibited enhanced chilling tolerance (**Table 1**), suggesting a negative function for this protein in chilling tolerance.

Through forward genetic screening, several genes have been identified as cold stress regulatory and responsive genes. Among the 16 genes we have identified as chilling tolerance genes, only *ACD6* and *ADS2* were previously shown to be freezing-responsive genes (Chen and Thelen, 2013). *AT4G12350* gene encoding ATMYB42 (**Table 2**), with a subtle cold stress-related phenotype, is a homologue of the ATMYB14/15 transcription factors that have been demonstrated to negatively regulate at least some cold stress response genes (Agarwal et al., 2006; Miura et al., 2007; Chen et al., 2013; Dong et al., 2021). It has been demonstrated that ATMYB42 is a regulator of phenylalanine and lignin biosynthesis (Geng et al., 2020).

Blast2GO analysis was conducted to understand the functional annotation of the 16 chilling tolerance genes (Conesa et al., 2005; BioBam Bioinformatics, 2019) (**Figure S6**; **Table S8**-**S10**). The 16 genes were grouped into 58 classes based on their biological processes, 17 classes according to their molecular functions, and 13 classes as cellular components or based on their subcellular locations suggesting that most, if not all, of the 16 chilling tolerance genes encode multiple functions (**Figure S6**; **Table S8**-**S10**).

The Kyoto Encyclopedia of Genes and Genomes (KEGG) (Kanehisa et al., 2017; BioBam Bioinformatics, 2019) pathway analyses of 25 genes, including nine genes with subtle chilling tolerance phenotypes, revealed that *AT2G18260* is involved in the pantothenate and CoA biosynthesis pathway (EC:2.7.7.3 in **Figure S7**), and *AT2G31360* in the biosynthesis of unsaturated fatty acids (EC:1.14.19.1 in **Figure S7**) (**Table 1**). The metabolome of Arabidopsis under temperature stress showed an increase in a small group of amine-containing metabolites (β‐alanine and putrescine) (Kaplan et al., 2004), and plants capable of cold acclimation accumulate polyunsaturates during cold stress. KEGG pathways analysis revealed that *AT2G27120* (**Table 2**) is involved in DNA replication, base/nucleotide excision repair, and purine metabolism pathways (EC:2.7.7.7 in **Figure S7**), *AT3G53520* (**Table 2**) is involved in amino sugar and nucleotide sugar metabolism pathways (EC:4.1.1.35 in **Figure S7**), and *AT5G54960* (**Table 2**) involved in glycolysis/gluconeogenesis (EC:4.1.1.1 in **Figure S7**). The connection between plant DNA damage response and responses to biotic and abiotic stresses has been reported (Nisa et al., 2019). Duplication of genes has been observed specifically for those involved in reproduction, DNA damage repair, and cold tolerance in the high-altitude plant *Eutrema heterophyllum* (Guo et al., 2018).

### MapMan analysis of chilling-responsive genes

The efficacy of the full-genome sequences for important crop species has been advanced by the development of detailed ontologies by programs such as MapMan (Usadel et al., 2009; Schwacke et al., 2019), which has assigned enzymes to over 1,200 groups covering almost all central metabolic pathways. Mapman facilitates analyses of large transcriptomic and proteomic datasets (Usadel et al., 2009). Though it was developed initially for analyses of Arabidopsis datasets (Usadel et al., 2009), MapMan ontology has been extended to several other species (Ling et al., 2013), including soybean (Nanjo et al., 2011), cotton (Christianson et al., 2010), maize (Usadel et al., 2009), potato (Kondrák et al., 2011), and tomato (Barsan et al., 2010). MapMan maps transcript profiling data onto pathways and genetic maps and generates response overlays that simplify the identification of shared features globally and on a gene-to-gene basis (Usadel et al., 2009; Pitzschke and Hirt, 2010). We conducted MapMan analyses of the identified 16 chilling tolerance genes (**Table 1**), and nine strong candidate genes (**Table 2**) using their transcript profiles (**Figures S3** & **Table S5**) and mapped 23 of the 25 genes to metabolism, biotic stress, cellular response, proteasome, autophagy and cellular function categories (**Figure S8**).

## Discussion

Genetic pathways regulating the expression of cold stress-responsive genes have been identified through either forward (Provart et al., 2016) or reverse genetics (Østergaard and Yanofsky, 2004; Alonso and Ecker, 2006; Chinnusamy et al., 2010; Provart et al., 2016). In *Arabidopsis*, changes in gene expression in response to cold stress are regulated by the C-REPEAT BINDING FACTOR (CBF)-mediated cold signaling pathway (Chinnusamy et al., 2010; Jeon and Kim, 2013). Cold stress elevates Ca^2+^ levels transiently and activates protein kinases, including MAP kinases, for cold acclimation (Lissarre et al., 2010). In transgenic Arabidopsis, CBF/DREB proteins overexpression led to desiccation and cold tolerance through ectopic expression of RD/COR genes (Jaglo-Ottosen et al., 1998; Kasuga et al., 1999). The transcription factors CBF (C-repeat-binding factor)/DREB1 (dehydration responsive element binding1) and ICE1 (inducer of CBF expression 1) have essential roles in regulating the expression of cold-responsive (COR) genes (Liu et al., 1998; Chinnusamy et al., 2010; Lissarre et al., 2010). The CBFs/DREBs induce several hundred genes by binding to their CRT/DRE elements (Vogel et al., 2005). REIL2 deficiency delays CBF/DREB regulon activation and reduces CBF/DREB protein accumulation in response to cold stress (Yu et al., 2020). Overexpression of a ribosomal biogenesis factor encoded by *STCH4*/*REIL2* enhances chilling and freezing tolerance in Arabidopsis (Yu et al., 2020). STCH4 presumably induces alterations in the ribosomal composition and functions at low temperatures to facilitate the translation of proteins required for plant development and survival under cold stress (Yu et al., 2020). Likewise, overexpression of genes encoding ice recrystallization inhibition (IRI) proteins LpIRI-a or LpIRI-b in Arabidopsis exhibited improved cell membrane stability in freezing and improved frost tolerance (Zhang et al., 2010). Open stomata 1 (OST1) protein kinase also plays a central role in regulating freezing tolerance in Arabidopsis, and its activity is regulated by a plasma membrane‐localized clade‐E growth‐regulating 2 (EGR2) phosphatase (Ding et al., 2019).

In this study, we investigated Arabidopsis natural variants collected from a broad geographical region to identify possible additional novel genetic mechanisms for chilling tolerance. A diverse collection of natural variants is a useful resource for identifying genetic mechanisms involved in various biological processes through GWAS. Arabidopsis is an ideal model plant distributed across 15.11 to 62.63 latitudes and -123.21 to 136.31 longitudes that include a diverse ecological range, including its ancestral Iberian Peninsula habitat to northern latitudes with an unknown glacial refugium (Alonso-Blanco et al., 2016). The 1,135 natural Arabidopsis variants collected from the diverse ecological ranges have been resequenced to facilitate identifying candidate genes for various traits through GWAS (Alonso-Blanco et al., 2016). The model plant Arabidopsis is particularly suitable for this study because of the large collection of mutants available to validate the candidate genes identified in GWAS (O’Malley et al., 2015).

The genetic basis of cold tolerance in numerous crops has been investigated using GWAS. For example, a GWAS for cold tolerance at the seedling stage among rice landraces discovered a total of 26 SNPs that were significantly associated with cold tolerance (Song et al., 2018). Similarly, GWAS and differentially expressed gene (DEG) analysis among germinating maize seeds revealed 30 SNPs and two DEGs associated with cold tolerance (Zhang et al., 2020). A GWAS among 200 cotton accessions collected from diverse geographical locations revealed an alcohol dehydrogenase gene (*GhSAD1*) associated with cold tolerance (Ge et al., 2021). In canola, GWAS led to the identification of 25 candidate genes that were previously shown to be associated with freezing tolerance, photosynthesis, or cold responsiveness in canola or Arabidopsis (Chao et al., 2021).

We developed a high-throughput phenotyping platform and determined the responses of seedlings of 417 of these ecotypes to chilling stress at 4 °C for 30 days. The 417 ecotypes showed a 10-fold difference in growth rate between the most chilling-sensitive and the most chilling-tolerant ecotypes. Therefore, this collection of natural variants was ideal for mining chilling tolerance genes (**Figure 2D**). To facilitate the identification of most of the chilling tolerance genes, we (i) phenotyped the ecotypes for responses to cold stress multiple times; and we used data from each experiment as well as the mean from all experiments to conduct GWAS. Two models, Linear Regression (LR) and EMMAX were assessed to accommodate population structures. We identified 33 candidate cold-responsive genes through GWAS (**Table S4**). Only 11 of the 33 genes were identified in GWAS when either LR or EMMAX model was used; 15 were identified in analyses with EMMAX and seven with the LR model (**Table 1**; **Table S4**).

Analyses of at least two independent insertion mutants for 29 of these genes identified 16 chilling tolerance genes (**Table 1**). Loss of function mutants of nine of the 16 chilling tolerance genes with reduced transcript levels under cold stress showed enhanced chilling tolerance, while mutants of four genes induced during cold stress showed increased sensitivity to prolonged cold stress (**Figure 2E**; **Table 1**). For three genes, an inverse relationship between the transcript levels and responses of mutants to cold stress was not observed. The inverse relationship between the growth of mutants and corresponding steady-state transcript levels of these 13 genes suggests that most of the 16 identified chilling tolerance genes are regulated at the transcriptional level for adapting Arabidopsis to cold stress. In addition to the 16 chilling tolerance genes, altered phenotypes for a single mutant of each of the nine genes were observed (**Table 2**). This suggests that these nine genes may subtly affect chilling tolerance. Investigation of additional mutants or complementation analyses of the loss-of-function mutants will establish the role of these nine putative chilling tolerance genes. As opposed to other GWAS, in our investigation we studied resequenced accessions (Alonso-Blanco et al., 2016). Therefore, we were able to precisely identify 33 strong candidate cold-responsive genes. Most importantly we further investigated 29 of these candidate genes for their possible role in cold tolerance through studying at least two insertion mutants (O’Malley et al., 2015). We identified 16 chilling tolerance genes based on altered mutant phenotypes in at least two insertion mutants. Furthermore, we also identify nine additional genes that may have subtle roles in chilling tolerance because only one of the insertion mutants for each of these genes showed altered chilling tolerance phenotypes. Our results are complementary to the results previously gathered through forward and reverse genetics. Only two of the 16 genes were previously identified.

Blast2GO analysis of all 16 chilling tolerance genes revealed that the 16 genes could be grouped into 58 classes based on the biological processes they are involved in (**Table 1**; **Figure S4**; **Table S8**-**S10**). This showed the complexity of cold tolerance mechanisms that interplay in adapting Arabidopsis to prolonged cold stress. We employed MapMan to map the identified 15 chilling-tolerance genes (**Table 1**) and eight of the nine strong candidate chilling-tolerance genes (**Table 2**) showing differential expression due to cold stress (**Figure S3**; **Table S5**) onto metabolism, biotic stress, cellular response, proteasome, autophagy and cellular function categories (**Figure S8**). MapMan analysis interestingly revealed the involvement of lipid metabolism (*ADS2*), biotic stress-related genes (*NB-ARC LRR*, *TIR-NB-LRR*, *AtRLP39*, *PER72*, *LRR protein kinase*), a protein involved in heat stress (DNAJ heat shock N-terminal domain-containing protein), ubiquitin and autophagy-dependent degradation, proteolysis (*EDA41*), vesicle transport and protein targeting (*AtSYP112*) and transcriptional regulation (*HMGB6*, stress-associated protein 7; *AtMYB42*) in response to cold stress (**Figure S8**).

Transcription factors are involved in regulating the expression of cold-responsive genes. For example, C-REPEAT BINDING FACTOR (CBF)-mediated cold signaling pathway (Chinnusamy et al., 2010; Jeon and Kim, 2013). The regulation of CBF genes plays a crucial role in the CBF-COR signaling pathway (Liu et al., 2019). The promoters of CBFs contain MYB recognition sequences suggesting MYB-related transcription factor participation in the cold induction of CBFs (Chinnusamy et al., 2003). We identified a strong candidate cold-tolerance gene encoding a MYB transcription factor, *AtMYB42*, a homologue of *MYB15*, which was shown to be involved in cold tolerance earlier (**Table 2**). Overexpression of *MYB15* resulted in decreased freezing tolerance, while its knock-out mutant displayed an improved freezing tolerance (Agarwal et al., 2006). In our study, a T-DNA insertion *atmyb42* mutant showed enhanced chilling tolerance (**Table 2**). MYB15 interacts physically with ICE1, which regulates the transcription of CBF genes in response to cold (Chinnusamy et al., 2003). Overexpression of ICE1 boosts the expression of the CBF regulon, thus improving freezing tolerance in transgenic plants (Chinnusamy et al., 2003).

Lipid metabolism plays a key role in response to cold stress (Barrero-Sicilia et al., 2017). In cold stress, one of the adaptive responses is re-modeling cell membrane fluidity, which is achieved by increasing the unsaturated fatty acid composition of membrane lipids (Upchurch, 2008). Transcriptomics analysis of amino acid catabolism established a link between cellular regulation and protein degradation in response to various environmental stresses, including cold stress (Kazemi-Shahandashti and Maali-Amiri, 2018). E3 ubiquitin ligases are involved with biotic and abiotic stresses, including cold stress in Arabidopsis (Dong et al., 2006; Suh and Kim, 2015) and rice (Byun et al., 2017; Xu and Xue, 2019). Our findings in the present study (**Figure S8**) also corroborate the earlier studies exhibiting the involvement of lipid metabolism, protein degradation, and the ubiquitin-proteasome system and DnaJ proteins in cold stress (Barrero-Sicilia et al., 2017; Kazemi-Shahandashti and Maali-Amiri, 2018; Xu and Xue, 2019). The DnaJ proteins are treated as common cellular stress sensors because of their expression by many factors such as heat, cold, and drought (Rajan and D’Silva, 2009; Liu and He, 2020).

The common pathways among cold stress and other abiotic and biotic stress signaling suggest the cross-talks among the pathways (Solanke and Sharma, 2008). The identification of five novel genes encoding LRR domain-containing proteins is worth noting. Temperature affects disease resistance by broadly influencing plant growth, regulating plant-pathogen interactions and defense responses mediated by several disease resistance (*R*) genes (Garrett et al., 2006). The role of NBS-LRR genes in freezing tolerance has been established (Huang et al., 2010; Yang et al., 2010; Zbierzak et al., 2013). NB-LRR receptor functions are known to be modulated by cold stress by integrating an alternative H2A.Z histone variant into nucleosomes (Alcázar and Parker, 2011). NLRs or NLR‐like proteins act as centers linking low‐temperature stress and salicylic acid (SA)‐dependent growth inhibition (Zbierzak et al., 2013). Over-expression of an LHY-CCA1-Like transcription factor SgRVE6 results in increased expression of 6 NB-LRR encoding genes associated with tobacco cold-tolerance, and it provides the transgenic tobaccos with higher tolerance to cold stress (Chen et al., 2020). Constant exposure to cold or low temperatures might result in the accumulation of SA and the suppression of development (Chen et al., 2015). The inactivation of a ubiquitin‐conjugating enzyme, UBC13, compromises cold‐responsive gene activation and causes elevated SA concentration and growth inhibition at low temperature. The phenotypes of the *ubc13* mutant are partially dependent on an NLR gene, *SNC1*, implying that UBC13 is engaged in NLR function during low‐temperature stress (Wang et al., 2019). The defense regulator genes *SAG101*, *EDS1*, and *PAD4* negatively regulate the freezing tolerance in Arabidopsis, partly by modulating SA and diacylglycerol (DAG) homeostasis (Chen et al., 2015). The diacylglycerol acyltransferase 1 (DGAT1) is highly cold-responsive, and SA downregulates its cold-responsive expression (Chen et al., 2015). DGAT1 catalyzes the final step in the triacylglycerol (TAG) assembly by acyl transfer from acyl-CoA to DAG. During cold acclimation, freezing-tolerant plants displayed higher DGAT1 expression, resulting in increased TAG accumulation in response to subsequent freezing (Chen et al., 2015). DAG metabolism is also believed to act downstream of defense regulator genes SAG101, EDS1, and PAD4 in the SA-associated cold signaling pathway (Chen et al., 2015). The chilling sensitive (*chs*) mutants, *chs2* and *chs3*, of genes encoding R proteins of the TIR-NB-LRR class exhibited accumulation of high SA levels, specifically under cold stress (Eremina et al., 2016). None of the three NBS-LRR genes identified showed any similarity to previously cloned NB-LRR genes involved in freezing tolerance. *AT1G61310* encoding an NBS-LRR protein has been annotated to be a disease-resistance-like protein. *AT5G41750* encoding a TIR-NB-LRR protein was previously shown to be a candidate for the *DM1* (*Dangerous Mix 1*) gene involved in autoimmunity and incompatibility response (Bomblies et al., 2007). It is becoming evident that NB-LRR proteins, LRR-kinase, and RLP may have a significant role in signaling cold tolerance pathways in plants. Several studies have indicated that abiotic stress signaling pathways overlap with the disease resistance signaling pathways (Lee and Yeom, 2015). Some of the NB-LRR-type *R* genes serve as non-immune receptors and are involved in signaling for plant development. When grown below 16°C, the Arabidopsis *chilling-sensitive 2* (*chs2*) mutant demonstrated temperature-sensitive growth abnormalities comparable to those detected during defense responses (Huang et al., 2010). A gain-of-function mutant allele of the *RPP4* gene was detected in the *chs2* mutant. The mutant allele increases chilling sensitivity and expression of pathogenesis-related (*PR*) genes, the production of hydrogen peroxide, and SA when the mutant is cultured at 16°C (Huang et al., 2010). The Arabidopsis *chs3* mutant exhibiting induction of defense responses showed stunted growth and chlorosis at 16°C (Yang et al., 2010). *CHS3* encodes a TNL-LIM-type NB-LRR *R* gene that regulates the freezing tolerance.

The assignment of chilling tolerance function to 14 of the 16 identified genes is surprising. The overlap between the chilling tolerance-related genes in the prior and this study was observed just for two genes. One possible explanation could be that chilling tolerance is regulated by a very complex process, and all components of this process are yet to be identified. We looked at the mutations that resulted in the mutant alleles for 16 genes involved in adapting the natural variants to the temperate climate of the northern hemisphere. For none of the genes, we observed a nonsense mutation. One possible reason is that the genes may have vital and multiple functions, as we have seen for many of the genes identified in this study. Natural selection shapes the expression levels or structure of the involved proteins or enzymes without compromising the other functions encoded by the genes during the generation of new functions for adapting plants to new environments or growing conditions. We observed that SNPs identified by GWAS were localized to 5’-UTRs of three genes, 3’-UTR of one, and introns of three genes (**Table 1**; **Table S4**). A synonymous mutation was detected in *AT4G12000*, which encodes a SNARE-associated Golgi protein. This gene is highly expressed during cold stress, and two knockout mutants for this gene clearly showed super-sensitivity to cold stress (**Table 1**; **Figure S3M**; **Figure S5F**). It is possible that the synonymous mutation could impact the transcription of this gene if it is localized to an unidentified *cis*-acting element for transcription. Transcriptional regulation through *cis*-elements localized to UTRs is well established (Srivastava et al., 2018; Rose, 2019). Introns can contain splicing-regulatory sequences to autoregulate alternate splicing and transcription regulation (Thomas et al., 2012). Synonymous mutations in the open reading frames may also cause structural changes in mRNAs leading to changes in protein translation efficiency (Li et al., 2019a).

## Conclusion

The GWAS, together with insertion mutant analyses, revealed 16 chilling tolerance genes and nine strong candidate chilling tolerance genes. It was surprising that only two of the 16 identified genes were previously identified. The 14 novel chilling tolerance genes identified in this investigation indicated that multiple genetic mechanisms are involved in manifesting chilling tolerance in Arabidopsis. Thus, chilling tolerance is a complex trait and is governed by many genetic mechanisms. In this natural variant study, it was evident that none of the polymorphisms assisting the identification of 16 chilling tolerance genes cause either knockout or nonsynonymous mutations affecting loss or altered gene function. These mutations presumably altered gene expression levels. It is possible that these genes encode multiple functions, some of which could be vital. Loss of function mutations in these genes presumably lack necessary fitness values and were selected out by natural selection. Therefore, nature shaped the expression of most of these genes without causing any changes to the protein structures to provide better adaptation to temperate climate. This study identified 14 novel genes including five that encode novel leucine-rich repeat domain-containing proteins, including three NBS-LRR proteins. The knowledge gained through identification of 14 novel chilling tolerance genes complementes the ongoing effort on understanding cold tolerance mechanisms and provides a strong base for developing chilling tolerant crop varieties that would well adapt well to cold stress, which is becoming frequent because of climate change and a serious threat to sustainable crop production.

## Data availability statement

The raw data supporting the conclusions of this article will be made available by the authors, without undue reservation.

## Author contributions

DKS collected and increased seeds, developed the phenomics platform, collected and analyzed the data, wrote the manuscript, and prepared the Figures and Tables. CH wrote the software for image analyses and reviewed and approved manuscript. MKB designed the experiments and edited and finalized the manuscript. All authors contributed to the article and approved the submitted version.

## References

[B1] Abràmoff M. D. Magalhães P. J. Ram S. J. (2004) Image processing with ImageJ. Available at: https://dspace.library.uu.nl/handle/1874/204900 (Accessed May 5, 2022).

[B2] Agarwal M. Hao Y. Kapoor A. Dong C. H. Fujii H. Zheng X.. (2006). A R2R3 type MYB transcription factor is involved in the cold regulation of CBF genes and in acquired freezing tolerance. J. Biol. Chem. 281, 37636–37645. doi: 10.1074/JBC.M605895200 17015446

[B3] Alcázar R. Parker J. E. (2011). The impact of temperature on balancing immune responsiveness and growth in arabidopsis. Trends Plant Sci. 16, 666–675. doi: 10.1016/j.tplants.2011.09.001 21963982

[B4] Allen E. Xie Z. Gustafson A. M. Sung G. H. Spatafora J. W. Carrington J. C. (2004). Evolution of microRNA genes by inverted duplication of target gene sequences in arabidopsis thaliana. Nat. Genet. 36, 1282–1290. doi: 10.1038/ng1478 15565108

[B5] Alonso-Blanco C. Andrade J. Becker C. Bemm F. Bergelson J. Borgwardt K. M.. (2016). 1,135 genomes reveal the global pattern of polymorphism in arabidopsis thaliana. Cell 166, 481–491. doi: 10.1016/j.cell.2016.05.063 27293186 PMC4949382

[B6] Alonso J. M. Ecker J. R. (2006). Moving forward in reverse: Genetic technologies to enable genome-wide phenomic screens in arabidopsis. Nat. Rev. Genet. 7, 524–536. doi: 10.1038/nrg1893 16755288

[B7] Alonso J. M. Stepanova A. N. Leisse T. J. Kim C. J. Chen H. Shinn P.. (2003). Genome-wide insertional mutagenesis of arabidopsis thaliana. Science 301, 653–657. doi: 10.1126/science.1086391 12893945

[B8] Bao Y.-M. Wang J.-F. Huang J. Zhang H.-S. (2008). Molecular cloning and characterization of a novel SNAP25-type protein gene OsSNAP32 in rice (Oryza sativa l.). Mol. Biol. Rep. 35, 145–152. doi: 10.1007/s11033-007-9064-8 17380428

[B9] Barah P. Jayavelu N. D. Rasmussen S. Nielsen H. B. Mundy J. Bones A. M. (2013). Genome-scale cold stress response regulatory networks in ten arabidopsis thalianaecotypes. BMC Genomics 14, 722. doi: 10.1186/1471-2164-14-722 24148294 PMC3829657

[B10] Barrero-Sicilia C. Silvestre S. Haslam R. P. Michaelson L. V. (2017). Lipid remodelling: Unravelling the response to cold stress in arabidopsis and its extremophile relative eutrema salsugineum. Plant Sci. 263, 194–200. doi: 10.1016/j.plantsci.2017.07.017 28818375 PMC5567406

[B11] Barsan C. Sanchez-Bel P. Rombaldi C. Egea I. Rossignol M. Kuntz M.. (2010). Characteristics of the tomato chromoplast revealed by proteomic analysis. J Exp Bot 61, 2413–2431. doi: 10.1093/JXB/ERQ070. 20363867

[B12] Bassham D. C. Blatt M. R. (2008). SNAREs: Cogs and coordinators in signaling and development. Plant Physiol. 147, 1504–1515. doi: 10.1104/pp.108.121129 18678742 PMC2492632

[B13] ben Saad R. Zouari N. ben Ramdhan W. Azaza J. Meynard D. Guiderdoni E.. (2010). Improved drought and salt stress tolerance in transgenic tobacco overexpressing a novel A20/AN1 zinc-finger “AlSAP” gene isolated from the halophyte grass aeluropus littoralis. Plant Mol. Biol. 72, 171–190. doi: 10.1007/S11103-009-9560-4 19838809

[B14] Berestovoy M. A. Pavlenko O. S. Goldenkova-Pavlova I. V. (2020). Plant fatty acid desaturases: Role in the life of plants and biotechnological potential. Biol. Bull. Rev. 10, 127–139. doi: 10.1134/s2079086420020024

[B15] BioBam Bioinformatics (2019) OmicsBox – bioinformatics made easy (Accessed March 3, 2019).

[B16] Bomblies K. Lempe J. Epple P. Warthmann N. Lanz C. Dangl J. L.. (2007). Autoimmune response as a mechanism for a dobzhansky-muller-type incompatibility syndrome in plants. PloS Biol. 5, 1962–1972. doi: 10.1371/journal.pbio.0050236 PMC1964774 17803357

[B17] Bruinsma J. (2003). World agriculture: towards 2015/2030: an FAO perspective. London: Taylor & Francis Group.

[B18] Byun M. Y. Cui L. H. Oh T. K. Jung Y.-J. Lee A. Park K. Y.. (2017). Homologous U-box E3 ubiquitin ligases OsPUB2 and OsPUB3 are involved in the positive regulation of low temperature stress response in rice (Oryza sativa l.). Front. Plant Sci. 8. doi: 10.3389/fpls.2017.00016 PMC5247461 28163713

[B19] Chao W. S. Horvath D. P. Stamm M. J. Anderson J. V. (2021). Genome-wide association mapping of freezing tolerance loci in canola (Brassica napus l.). Agronomy 11, 233. doi: 10.3390/agronomy11020233

[B20] Chen Y. Chen Z. Kang J. Kang D. Gu H. Qin G. (2013). AtMYB14 regulates cold tolerance in arabidopsis. Plant Mol. Biol. Rep. 31, 87–97. doi: 10.1007/s11105-012-0481-z PMC3881570 24415840

[B21] Chen S. Huang H.-A. Chen J.-H. Fu C.-C. Zhan P.-L. Ke S.-W.. (2020). SgRVE6, a LHY-CCA1-Like transcription factor from fine-stem stylo, upregulates NB-LRR gene expression and enhances cold tolerance in tobacco. Front. Plant Sci. 11. doi: 10.3389/fpls.2020.01276 PMC7466579 32973836

[B22] Chen M. Thelen J. J. (2013). ACYL-LIPID DESATURASE2 is required for chilling and freezing tolerance in arabidopsis. Plant Cell 25, 1430–1444. doi: 10.1105/tpc.113.111179 23585650 PMC3663278

[B23] Chen M. Thelen J. J. (2016). *Acyl-lipid desaturase 1* primes cold acclimation response in *Arabidopsis* . Physiol. Plant 158, 11–22. doi: 10.1111/ppl.12448 27062193

[B24] Chen Q. F. Xu L. Tan W. J. Chen L. Qi H. Xie L. J.. (2015). Disruption of the arabidopsis defense regulator genes SAG101, EDS1, and PAD4 confers enhanced freezing tolerance. Mol. Plant 8, 1536–1549. doi: 10.1016/j.molp.2015.06.009 26149542 PMC5321072

[B25] Chepyshko H. Lai C.-P. Huang L.-M. Liu J.-H. Shaw J.-F. (2012). Multifunctionality and diversity of GDSL esterase/lipase gene family in rice (Oryza sativa l. japonica) genome: new insights from bioinformatics analysis. BMC Genomics 13, 309. doi: 10.1186/1471-2164-13-309 22793791 PMC3412167

[B26] Chinnusamy V. Ohta M. Kanrar S. Lee B. Hong X. Agarwal M.. (2003). ICE1: a regulator of cold-induced transcriptome and freezing tolerance in arabidopsis. Genes Dev. 17 (1043), 1043–1054. doi: 10.1101/GAD.1077503 PMC196034 12672693

[B27] Chinnusamy V. Zhu J. K. Sunkar R. (2010). Gene regulation during cold stress acclimation in plants. Methods Mol. Biol. 639, 39–55. doi: 10.1007/978-1-60761-702-0_3 20387039 PMC3064467

[B28] Christians M. J. Gingerich D. J. Hua Z. Lauer T. D. Vierstra R. D. (2012). The light-response BTB1 and BTB2 proteins assemble nuclear ubiquitin ligases that modify phytochrome b and d signaling in arabidopsis. Plant Physiol. 160, 118–134. doi: 10.1104/pp.112.199109 22732244 PMC3440189

[B29] Christianson J. A. Llewellyn D. J. Dennis E. S. Wilson I. W. (2010). Global Gene Expression Responses to Waterlogging in Roots and Leaves of Cotton (Gossypium hirsutum L.). Plant Cell Physiol 51, 21–37. doi: 10.1093/PCP/PCP163. 19923201

[B30] Ciais P. Reichstein M. Viovy N. Granier A. Ogée J. Allard V.. (2005). Europe-Wide reduction in primary productivity caused by the heat and drought in 2003. Nature 437, 529–533. doi: 10.1038/nature03972 16177786

[B31] Conesa A. Götz S. García-Gómez J. M. Terol J. Talón M. Robles M. (2005). Blast2GO: A universal tool for annotation, visualization and analysis in functional genomics research. Bioinformatics 21, 3674–3676. doi: 10.1093/bioinformatics/bti610 16081474

[B32] Ding Y. Lv J. Shi Y. Gao J. Hua J. Song C.. (2019). EGR 2 phosphatase regulates OST 1 kinase activity and freezing tolerance in arabidopsis. EMBO J. 38, e99819. doi: 10.15252/embj.201899819 30429206 PMC6315290

[B33] Dixit A. R. Dhankher O. P. (2011). A novel stress-associated protein ‘AtSAP10’ from arabidopsis thaliana confers tolerance to nickel, manganese, zinc, and high temperature stress. PloS One 6, e20921. doi: 10.1371/journal.pone.0020921 21695274 PMC3111467

[B34] Dong C.-H. Agarwal M. Zhang Y. Xie Q. Zhu J.-K. (2006). The negative regulator of plant cold responses, HOS1, is a RING E3 ligase that mediates the ubiquitination and degradation of ICE1. Proc. Natl. Acad. Sci. 103, 8281–8286. doi: 10.1073/PNAS.0602874103 16702557 PMC1472463

[B35] Dong J. Cao L. Zhang X. Zhang W. Yang T. Zhang J.. (2021). An R2R3-MYB transcription factor RmMYB108 responds to chilling stress of Rosa multiflora and conferred cold tolerance of arabidopsis. Front. Plant Sci. 12(1525). doi: 10.3389/FPLS.2021.696919/BIBTEX PMC8353178 34386027

[B36] Eremina M. Rozhon W. Poppenberger B. (2016). Hormonal control of cold stress responses in plants. Cell. Mol. Life Sci. 73, 797–810. doi: 10.1007/s00018-015-2089-6 26598281 PMC11108489

[B37] Fournier-Level A. Korte A. Cooper M. D. Nordborg M. Schmitt J. Wilczek A. M. (2011). A map of local adaptation in arabidopsis thaliana. Science 334, 86–89. doi: 10.1126/science.1209271 21980109

[B38] Garrett K. A. Dendy S. P. Frank E. E. Rouse M. N. Travers S. E. (2006). Climate change effects on plant disease: Genomes to ecosystems. Annu. Rev. Phytopathol. 44, 489–509. doi: 10.1146/annurev.phyto.44.070505.143420 16722808

[B39] Geng P. Zhang S. Liu J. Zhao C. Wu J. Cao Y.. (2020). MYB20, MYB42, MYB43, and MYB85 regulate phenylalanine and lignin biosynthesis during secondary cell wall formation1[OPEN]. Plant Physiol. 182, 1272–1283. doi: 10.1104/PP.19.01070 31871072 PMC7054866

[B40] Ge C. Wang L. Yang Y. Liu R. Liu S. Chen J.. (2021). Genome-wide association study identifies variants of GhSAD1 conferring cold tolerance in cotton. J. Exp. Bot. 73 (7), 2222–2237. doi: 10.1093/JXB/ERAB555 34919655

[B41] Gingerich D. J. Gagne J. M. Salter D. W. Hellmann H. Estelle M. Ma L.. (2005). Cullins 3a and 3b assemble with members of the broad complex/tramtrack/bric-a-brac (BTB) protein family to form essential ubiquitin-protein ligases (E3s) in arabidopsis. J. Biol. Chem. 280, 18810–18821. doi: 10.1074/jbc.M413247200 15749712

[B42] Grasser K. D. Grill S. Duroux M. Launholt D. Thomsen M. S. Nielsen B. V.. (2004). HMGB6 from arabidopsis thaliana specifies a novel type of plant chromosomal HMGB protein. Biochemistry 43, 1309–1314. doi: 10.1021/bi035931c 14756567

[B43] Grimm D. G. Roqueiro D. Salomé P. A. Kleeberger S. Greshake B. Zhu W.. (2017). easyGWAS: A cloud-based platform for comparing the results of genome-wide association studies. Plant Cell 29, 5–19. doi: 10.1105/tpc.16.00551 27986896 PMC5304348

[B44] Guo X. Hu Q. Hao G. Wang X. Zhang D. Ma T.. (2018). The genomes of two eutrema species provide insight into plant adaptation to high altitudes. DNA Res. 25, 307–315. doi: 10.1093/dnares/dsy003 29394339 PMC6014361

[B45] Guo M. Liu X. Jiang Y. Yu J. Meng T. (2021). Identification of arabidopsis genes associated with cold tolerance based on integrated bioinformatics analysis. J. Plant Interact. 16 (1), pp.344–pp.353. doi: 10.1080/17429145.2021.1955164

[B46] Hancock A. M. Brachi B. Faure N. Horton M. W. Jarymowycz L. B. Sperone F. G.. (2011). Adaptation to climate across the arabidopsis thaliana genome. Sci. 334, 83–86. doi: 10.1126/science.1209244 21980108

[B47] Hannah M. A. Heyer A. G. Hincha D. K. (2005). A global survey of gene regulation during cold acclimation in arabidopsis thaliana. PloS Genet. 1, e26. doi: 10.1371/journal.pgen.0010026 16121258 PMC1189076

[B48] Hannah M. A. Wiese D. Freund S. Fiehn O. Heyer A. G. Hincha D. K. (2006). Natural genetic variation of freezing tolerance in arabidopsis. Plant Physiol. 142 (1), pp.98–pp112. doi: 10.1104/pp.106.081141 PMC1557609 16844837

[B49] Hong J. K. Choi H. W. Hwang I. S. Kim D. S. Kim N. H. Choi D. S.. (2008). Function of a novel GDSL-type pepper lipase gene, CaGLIP1, in disease susceptibility and abiotic stress tolerance. Planta 227, 539–558. doi: 10.1007/s00425-007-0637-5 17929052

[B50] Huang X. Li J. Bao F. Zhang X. Yang S. (2010). A gain-of-function mutation in the arabidopsis disease resistance gene RPP4 confers sensitivity to low temperature. Plant Physiol. 154, 796–809. doi: 10.1104/pp.110.157610 20699401 PMC2949010

[B51] Huang J. Wang M. M. Jiang Y. Bao Y. M. Huang X. Sun H.. (2008). Expression analysis of rice A20/AN1-type zinc finger genes and characterization of ZFP177 that contributes to temperature stress tolerance. Gene 420, 135–144. doi: 10.1016/j.gene.2008.05.019 18588956

[B52] Hu X. Kong X. Wang C. Ma L. Zhao J. Wei J.. (2014). Proteasome-mediated degradation of FRIGIDA modulates flowering time in arabidopsis during vernalization. Plant Cell 26, 4763–4781. doi: 10.1105/tpc.114.132738 25538183 PMC4311208

[B53] Hwang I. S. Choi D. S. Kim N. H. Kim D. S. Hwang B (2014). The pepper cysteine/histidine-rich DC1 domain protein CaDC1 binds both RNA and DNA and is required for plant cell death and defense response. K. New Phytol. 201, 518–530. doi: 10.1111/nph.12521 24117868

[B54] Ihaka R. Gentleman R. (1996). R: A language for data analysis and graphics. J. Comput. Graphical Stat 5, 299–314. doi: 10.1080/10618600.1996.10474713

[B55] Jaglo-Ottosen K. R. Gilmour S. J. Zarka D. G. Schabenberger O. Thomashow M. F. (1998). Arabidopsis CBF1 overexpression induces COR genes and enhances freezing tolerance. Science 280, 104–106. doi: 10.1126/science.280.5360.104 9525853

[B56] Jeon J. Kim J. (2013). Cold stress signaling networks in arabidopsis. J. Plant Biol. 56, 69–76. doi: 10.1007/s12374-013-0903-y

[B57] Kaler A. S. Purcell L. C. (2019). Estimation of a significance threshold for genome-wide association studies. BMC Genomics 20, 618. doi: 10.1186/S12864-019-5992-7 31357925 PMC6664749

[B58] Kanehisa M. Furumichi M. Tanabe M. Sato Y. Morishima K. (2017). KEGG: New perspectives on genomes, pathways, diseases and drugs. Nucleic Acids Res. 45, D353–D361. doi: 10.1093/nar/gkw1092 27899662 PMC5210567

[B59] Kang H. M. Sul J. H. Service S. K. Zaitlen N. A. Kong S. Y. Freimer N. B.. (2010). Variance component model to account for sample structure in genome-wide association studies. Nat. Genet. 42, 348–354. doi: 10.1038/NG.548 20208533 PMC3092069

[B60] Kanneganti V. Gupta A. K. (2008). Overexpression of OsiSAP8, a member of stress associated protein (SAP) gene family of rice confers tolerance to salt, drought and cold stress in transgenic tobacco and rice. Plant Mol. Biol. 66, 445–462. doi: 10.1007/S11103-007-9284-2 18205020

[B61] Kaplan F. Kopka J. Haskell D. W. Zhao W. Schiller K. C. Gatzke N.. (2004). Exploring the temperature-stress metabolome of arabidopsis. Plant Physiol. 136, 4159–4168. doi: 10.1104/pp.104.052142 15557093 PMC535846

[B62] Kasuga M. Liu Q. Miura S. Yamaguchi-Shinozaki K. Shinozaki K. (1999). Improving plant drought, salt, and freezing tolerance by gene transfer of a single stress-inducible transcription factor. Nat. Biotechnol. 17, 287–291. doi: 10.1038/7036 10096298

[B63] Kazemi-Shahandashti S. S. Maali-Amiri R. (2018). Global insights of protein responses to cold stress in plants: Signaling, defence, and degradation. J. Plant Physiol. 226, 123–135. doi: 10.1016/J.JPLPH.2018.03.022 29758377

[B64] Kondrák M. Marincs F. Kalapos B. Juhász Z. Bánfalvi Z. (2011). Transcriptome Analysis of Potato Leaves Expressing the Trehalose-6-Phosphate Synthase 1 Gene of Yeast. PLoS One 6, 23466. doi: 10.1371/JOURNAL.PONE.0023466. PMC3156770 21858131

[B65] Kwak K. J. Kim J. Y. Kim Y. O. Kang H. (2007). Characterization of transgenic arabidopsis plants overexpressing high mobility group b proteins under high salinity, drought or cold stress. Plant Cell Physiol. 48, 221–231. doi: 10.1093/pcp/pcl057 17169924

[B66] Kwon S. J. Jin H. C. Lee S. Nam M. H. Chung J. H. Kwon S. I.. (2009). GDSL lipase-like 1 regulates systemic resistance associated with ethylene signaling in arabidopsis. Plant J. 58, 235–245. doi: 10.1111/j.1365-313X.2008.03772.x 19077166

[B67] Lee H. A. Yeom S. I. (2015). Plant NB-LRR proteins: tightly regulated sensors in a complex manner. Brief Funct. Genomics 14, 233–242. doi: 10.1093/BFGP/ELV012 25825425

[B68] Lekeux G. Crowet J. M. Nouet C. Joris M. Jadoul A. Bosman B.. (2019). Homology modeling and *in vivo* functional characterization of the zinc permeation pathway in a heavy metal p-type ATPase. J. Exp. Bot. 70, 329–341. doi: 10.1093/jxb/ery353 30418580 PMC6305203

[B69] Lekeux G. Laurent C. Joris M. Jadoul A. Jiang D. Bosman B.. (2018). Di-cysteine motifs in the c-terminus of plant HMA4 proteins confer nanomolar affinity for zinc and are essential for HMA4 function *in vivo* . J. Exp. Bot. 69, 5547–5560. doi: 10.1093/jxb/ery311 30137564 PMC6255694

[B70] Leviatan N. Alkan N. Leshkowitz D. Fluhr R. (2013). Genome-wide survey of cold stress regulated alternative splicing in *Arabidopsis thaliana* with tiling microarray. PloS One 8, e66511. doi: 10.1371/journal.pone.0066511 23776682 PMC3679080

[B71] Li J. J. Chew G. L. Biggin M. D. (2019a). Quantitative principles of cis-translational control by general mRNA sequence features in eukaryotes. Genome Biol. 20, 162. doi: 10.1186/s13059-019-1761-9 31399036 PMC6689182

[B72] Li Y. Guan K. Schnitkey G. D. DeLucia E. Peng B. (2019b). Excessive rainfall leads to maize yield loss of a comparable magnitude to extreme drought in the united states. Glob. Chang Biol. 25, gcb.14628. doi: 10.1111/gcb.14628 PMC6850578 31033107

[B73] Ling M. H. T. Rabara R. C. Tripathi P. Rushton P. J. Ge X. (2013). Extending MapMan Ontology to Tobacco for Visualization of Gene Expression. Dataset Pap Biol 2013, 1–7. doi: 10.7167/2013/706465. PMC3583537 23457664

[B74] Lissarre M. Ohta M. Sato A. Miura K. (2010). Plant signaling & behavior cold-responsive gene regulation during cold acclimation in plants. Taylor Francis 5, 948–952. doi: 10.4161/psb.5.8.12135 PMC3115169 20699657

[B75] Liu Y. Dang P. Liu L. He C. (2019). Cold acclimation by the CBF–COR pathway in a changing climate: Lessons from arabidopsis thaliana. Plant Cell Rep. 38, 511. doi: 10.1007/S00299-019-02376-3 30652229 PMC6488690

[B76] Liu J. He Z. (2020). Small DNA methylation, big player in plant abiotic stress responses and memory. Front. Plant Sci. 11(1977). doi: 10.3389/FPLS.2020.595603/BIBTEX PMC7758401 33362826

[B77] Liu Q. Kasuga M. Sakuma Y. Abe H. Miura S. Yamaguchi-Shinozaki K.. (1998). Two transcription factors, DREB1 and DREB2, with an EREBP/AP2 DNA binding domain separate two cellular signal transduction pathways in drought- and low-temperature-responsive gene expression, respectively, in arabidopsis. Plant Cell 10, 1391–1406. doi: 10.1105/tpc.10.8.1391 9707537 PMC144379

[B78] Lu H. Salimian S. Gamelin E. Wang G. Fedorowski J. LaCourse W.. (2009). Genetic analysis of *acd6-1* reveals complex defense networks and leads to identification of novel defense genes in arabidopsis. Plant J. 58, 401–412. doi: 10.1111/j.1365-313X.2009.03791.x 19144005 PMC2727925

[B79] Lyons J. M. Breidenbach R. W. (1981). Responses of plants to environmental stresses. j. levitt, t. t. kozlowski. Q Rev. Biol. 56, 480–481. doi: 10.1086/412488

[B80] Manacorda C. A. Asurmendi S. (2018). Arabidopsis phenotyping through geometric morphometrics. Gigascience 7, 1–20. doi: 10.1093/gigascience/giy073 PMC6041757 29917076

[B81] Meyer C.-L. Pauwels M. Briset L. Godé C. Salis P. Bourceaux A.. (2016). Potential preadaptation to anthropogenic pollution: evidence from a common quantitative trait locus for zinc and cadmium tolerance in metallicolous and nonmetallicolous accessions of *Arabidopsis halleri* . New Phytol. 212, 934–943. doi: 10.1111/nph.14093 27504589

[B82] Miura K. Jing B. J. Lee J. Chan Y. Y. Stirm V. Miura T.. (2007). SIZ1-mediated sumoylation of ICE1 controls CBF3/DREB1A expression and freezing tolerance in arabidopsis. Plant Cell 19, 1403–1414. doi: 10.1105/tpc.106.048397 17416732 PMC1913760

[B83] Miura K. Ohta M. (2010). SIZ1, a small ubiquitin-related modifier ligase, controls cold signaling through regulation of salicylic acid accumulation. J. Plant Physiol. 167, 555–560. doi: 10.1016/j.jplph.2009.11.003 19959255

[B84] Mukhopadhyay A. Vij S. Tyagi A. K. (2004). Overexpression of a zinc-finger protein gene from rice confers tolerance to cold, dehydration, and salt stress in transgenic tobacco. Proc. Natl. Acad. Sci. 101 (16), 6309–6314. doi: 10.1073/pnas.0401572101 PMC395965 15079051

[B85] Nanjo Y. Skultety L. Uváčková L. Klubicová K. Hajduch M. Komatsu S. (2012). Mass spectrometry-based analysis of proteomic changes in the root tipsof flooded soybean seedlings. J Proteome Res 11, 372–385. doi: 10.1021/PR200701Y 22136409

[B86] Nisa M. U. Huang Y. Benhamed M. Raynaud C. (2019). The plant DNA damage response: Signaling pathways leading to growth inhibition and putative role in response to stress conditions. Front. Plant Sci. 10. doi: 10.3389/fpls.2019.00653 PMC6534066 31164899

[B87] Østergaard L. Yanofsky M. F. (2004). Establishing gene function by mutagenesis in arabidopsis thaliana. Plant J. 39, 682–696. doi: 10.1111/j.1365-313X.2004.02149.x 15315632

[B88] O’Malley R. C. Barragan C. C. Ecker J. R. (2015). A user’s guide to the Arabidopsis T-DNA insertion mutant collections. Methods Mol Biol 1284, 323–342. doi: 10.1007/978-1-4939-2444-8_16 25757780 PMC5215775

[B89] O’Malley R. C. Ecker J. R. (2010). Linking genotype to phenotype using the arabidopsis unimutant collection. Plant J. 61, 928–940. doi: 10.1111/j.1365-313X.2010.04119.x 20409268

[B90] Palusa S. G. Ali G. S. Reddy A. S. N. (2007). Alternative splicing of pre-mRNAs of arabidopsis serine/arginine-rich proteins: Regulation by hormones and stresses. Plant J. 49, 1091–1107. doi: 10.1111/j.1365-313X.2006.03020.x 17319848

[B91] Pedersen D. S. Grasser K. D. (2010). The role of chromosomal HMGB proteins in plants. Biochim. Biophys. Acta Gene Regul. Mech. 1799, 171–174. doi: 10.1016/j.bbagrm.2009.11.004 20123078

[B92] Pitzschke A. Hirt H. (2010). Bioinformatic and Systems Biology Tools to Generate Testable Models of Signaling Pathways and Their Targets. Plant Physiol 152, 460. doi: 10.1104/PP.109.149583. 19915012 PMC2815901

[B93] Provart N. J. Alonso J. Assmann S. M. Bergmann D. Brady S. M. Brkljacic J.. (2016). 50 years of arabidopsis research: highlights and future directions. New Phytol. 209, 921–944. doi: 10.1111/nph.13687 26465351

[B94] Rajan V. B. D’Silva P. (2009). Arabidopsis thaliana J-class heat shock proteins: Cellular stress sensors. Funct. Integr. Genomics 9, 433–446. doi: 10.1007/S10142-009-0132-0/FIGURES/5 19633874

[B95] Rathan N. D. Krishna H. Ellur R. K. Sehgal D. Govindan V. Ahlawat A. K.. (2022). Genome-wide association study identifies loci and candidate genes for grain micronutrients and quality traits in wheat (*Triticum aestivum* l.). Sci. Rep. 12 (1), 1–15. doi: 10.1038/s41598-022-10618-w 35487909 PMC9054743

[B96] Rose A. B. (2019). Introns as gene regulators: A brick on the accelerator. Front. Genet. 10. doi: 10.3389/fgene.2018.00672 PMC6374622 30792737

[B97] Rosenzweig C. Tubiello F. N. Goldberg R. Mills E. Bloomfield J. (2002). Increased crop damage in the US from excess precipitation under climate change. Global Environ. Change 12, 197–202. doi: 10.1016/S0959-3780(02)00008-0

[B98] Sahoo D. K. Borcherding D. C. Chandra L. Jergens A. E. Atherly T. Bourgois-Mochel A.. (2022). Differential transcriptomic profiles following stimulation with lipopolysaccharide in intestinal organoids from dogs with inflammatory bowel disease and intestinal mast cell tumor. Cancers (Basel) 14, 3525. doi: 10.3390/CANCERS14143525/S1 35884586 PMC9322748

[B99] Sánchez J. L. Fraile R. de la Madrid J. L. de la Fuente M. T. Rodríguez P. Castro A. (1996). Crop damage: The hail size factor. J. Appl. Meteorol. 35, 1535–1541. doi: 10.1175/1520-0450(1996)035<1535:CDTHSF>2.0.CO;2

[B100] Sanderfoot A. A. Assaad F. F. Raikhel N. V. (2000). The arabidopsis genome. an abundance of soluble n-ethylmaleimide-sensitive factor adaptor protein receptors. Plant Physiol. 124, 1558–1569. doi: 10.1104/pp.124.4.1558 11115874 PMC59855

[B101] Sanghera G. S. Wani S. H. Hussain W. Singh N. B. (2011). Engineering cold stress tolerance in crop plants. Curr. Genomics 12, 30–43. doi: 10.2174/138920211794520178 21886453 PMC3129041

[B102] Schindelin J. Arganda-Carreras I. Frise E. Kaynig V. Longair M. Pietzsch T.. (2012). Fiji: An open-source platform for biological-image analysis. Nat. Methods 9, 676–682. doi: 10.1038/nmeth.2019 22743772 PMC3855844

[B103] Schwacke R. Ponce-Soto G. Y. Krause K. Bolger A. M. Arsova B. Hallab A.. (2019). MapMan4: A Refined Protein Classification and Annotation Framework Applicable to Multi-Omics Data Analysis. Mol Plant 12, 879–892. doi: 10.1016/J.MOLP.2019.01.003 30639314

[B104] Shakiba M. H. Ali M. S. M. Rahman R. N. Z. R. A. Salleh A. B. Leow T. C. (2016). Cloning, expression and characterization of a novel cold-adapted GDSL family esterase from photobacterium sp. strain J15. Extremophiles 20, 45–55. doi: 10.1007/s00792-015-0796-4 26475626

[B105] Solanke A. U. Sharma A. K. (2008). Signal transduction during cold stress in plants. Physiol. Mol. Biol. Plants 14, 69. doi: 10.1007/S12298-008-0006-2 23572874 PMC3550661

[B106] Song J. Li J. Sun J. Hu T. Wu A. Liu S.. (2018). Genome-wide association mapping for cold tolerance in a core collection of rice (Oryza sativa l.) landraces by using high-density single nucleotide polymorphism markers from specific-locus amplified fragment sequencing. Front. Plant Sci. 9. doi: 10.3389/FPLS.2018.00875/BIBTEX PMC6036282 30013584

[B107] Srivastava A. K. Lu Y. Zinta G. Lang Z. Zhu J. K. (2018). UTR-dependent control of gene expression in plants. Trends Plant Sci. 23, 248–259. doi: 10.1016/j.tplants.2017.11.003 29223924 PMC5828884

[B108] Ströher E. Wang X. Roloff N. Klein P. Husemann A. Dietz K.-J. (2009). Redox-dependent regulation of the stress-induced zinc-finger protein SAP12 in arabidopsis thaliana. Mol. Plant 2, 357–367. doi: 10.1093/mp/ssn084 19825620

[B109] Suh J. Y. Kim W. T. (2015). Arabidopsis RING E3 ubiquitin ligase AtATL80 is negatively involved in phosphate mobilization and cold stress response in sufficient phosphate growth conditions. Biochem. Biophys. Res. Commun. 463, 793–799. doi: 10.1016/J.BBRC.2015.06.015 26086094

[B110] Sun X.-H. Yu G. Li J.-T. Jia P. Zhang J.-C. Jia C.-G.. (2014). A heavy metal-associated protein (AcHMA1) from the halophyte, atriplex canescens (Pursh) nutt., confers tolerance to iron and other abiotic stresses when expressed in saccharomyces cerevisiae. Int. J. Mol. Sci. 15, 14891–14906. doi: 10.3390/ijms150814891 25153638 PMC4159888

[B111] Thomashow M. F. (1998). Role of cold-responsive genes in plant freezing tolerance. Plant Physiol 118, 1–8. doi: 10.1104/PP.118.1.1 PMC1539187 9733520

[B112] Thomas J. Palusa S. G. Prasad K. V. S. K. Ali G. S. Surabhi G.-K. Ben-Hur A.. (2012). Identification of an intronic splicing regulatory element involved in auto-regulation of alternative splicing of *SCL33* pre-mRNA. Plant J. 72, 935–946. doi: 10.1111/tpj.12004 22913769

[B113] Todesco M. Balasubramanian S. Hu T. T. Traw M. B. Horton M. Epple P.. (2010). Natural allelic variation underlying a major fitness trade-off in arabidopsis thaliana. Nature 465, 632–636. doi: 10.1038/nature09083 20520716 PMC3055268

[B114] Uemura T. Ueda T. Ohniwa R. L. Nakano A. Takeyasu K. Sato M. H. (2004). Systematic analysis of SNARE molecules in Arabidopsis: dissection of the post-Golgi network in plant cells. Cell Struct Funct 29, 49–65. doi: 10.1247/csf.29.49 15342965

[B115] Upchurch R. G. (2008). Fatty acid unsaturation, mobilization, and regulation in the response of plants to stress. Biotechnol. Lett. 30, 967–977. doi: 10.1007/s10529-008-9639-z 18227974

[B116] Usadel B. Poree F. Nagel A. Lohse M. Czedik-Eysenberg A. Stitt M. (2009). A guide to using MapMan to visualize and compare omics data in plants: A case study in the crop species, maize. Plant Cell Environ. 32, 1211–1229. doi: 10.1111/j.1365-3040.2009.01978.x 19389052

[B117] van der Velde M. Wriedt G. Bouraoui F. (2010). Estimating irrigation use and effects on maize yield during the 2003 heatwave in France. Agric. Ecosyst. Environ. 135, 90–97. doi: 10.1016/j.agee.2009.08.017

[B118] Vasseur F. Exposito-Alonso M. Ayala-Garay O. J. Wang G. Enquist B. J. Vile D.. (2018). Adaptive diversification of growth allometry in the plant arabidopsis thaliana. Proc. Natl. Acad. Sci. U.S.A. 115, 3416–3421. doi: 10.1073/pnas.1709141115 29540570 PMC5879651

[B119] Vij S. Tyagi A. K. (2006). Genome-wide analysis of the stress associated protein (SAP) gene family containing A20/AN1 zinc-finger(s) in rice and their phylogenetic relationship with arabidopsis. Mol. Genet. Genomics 276, 565–575. doi: 10.1007/s00438-006-0165-1 17033811

[B120] Vogel J. T. Zarka D. G. Van Buskirk H. A. Fowler S. G. Thomashow M. F. (2005). Roles of the CBF2 and ZAT12 transcription factors in configuring the low temperature transcriptome of arabidopsis. Plant J. 41, 195–211. doi: 10.1111/j.1365-313X.2004.02288.x 15634197

[B121] Wang L. Wen R. Wang J. Xiang D. Wang Q. Zang Y.. (2019). Arabidopsis UBC13 differentially regulates two programmed cell death pathways in responses to pathogen and low-temperature stress. New Phytol. 221, 919–934. doi: 10.1111/nph.15435 30218535

[B122] Wang T. Xue X. Xie X. Ye K. Zhu X. Elston R. C. (2018). Adjustment for covariates using summary statistics in GWAS analysis. Genet. Epidemiol. 42, 812. doi: 10.1002/GEPI.22148 30238496 PMC6239891

[B123] Winter D. Vinegar B. Nahal H. Ammar R. Wilson G. V. Provart N. J. (2007). An “Electronic fluorescent pictograph” browser for exploring and analyzing Large-scale biological data sets. PloS One 2, e718. doi: 10.1371/journal.pone.0000718 17684564 PMC1934936

[B124] Xie H. Sun Y. Cheng B. Xue S. Cheng D. Liu L.. (2019). Variation in ICE1 methylation primarily determines phenotypic variation in freezing tolerance in arabidopsis thaliana. Plant Cell Physiol. 60, 152–165. doi: 10.1093/pcp/pcy197 30295898

[B125] Xiong F. Ren J. J. Yu Q. Wang Y. Y. Kong L. J. Otegui M. S.. (2019). AtBUD13 affects pre-mRNA splicing and is essential for embryo development in arabidopsis. Plant J. 98, 714–726. doi: 10.1111/tpj.14268 30720904

[B126] Xu Y. Berkowitz O. Narsai R. De Clercq I. Hooi M. Bulone V.. (2019). Mitochondrial function modulates touch signalling in *Arabidopsis thaliana* . Plant J. 97, 623–645. doi: 10.1111/tpj.14183 30537160

[B127] Xu F. Q. Xue H. W. (2019). The ubiquitin-proteasome system in plant responses to environments. Plant Cell Environ. 42, 2931–2944. doi: 10.1111/pce.13633 31364170

[B128] Yamaguchi-Shinozaki K. Shinozaki K. (2006). Transcriptional regulatory networks in cellular responses and tolerance to dehydration and cold stresses. Annu. Rev. Plant Biol. 57, 781–803. doi: 10.1146/annurev.arplant.57.032905.105444 16669782

[B129] Yang H. Shi Y. Liu J. Guo L. Zhang X. Yang S. (2010). A mutant CHS3 protein with TIR-NB-LRR-LIM domains modulates growth, cell death and freezing tolerance in a temperature-dependent manner in arabidopsis. Plant J. 63, 283–296. doi: 10.1111/j.1365-313X.2010.04241.x 20444230

[B130] Yu H. Kong X. Huang H. Wu W. Park J. Yun D. J.. (2020). STCH4/REIL2 confers cold stress tolerance in arabidopsis by promoting rRNA processing and CBF protein translation. Cell Rep. 30, 229–242.e5. doi: 10.1016/j.celrep.2019.12.012 31914389

[B131] Zbierzak A. M. Porfirova S. Griebel T. Melzer M. Parker J. E. Dörmann P. (2013). A TIR-NBS protein encoded by arabidopsis *Chilling sensitive 1* (*CHS1*) limits chloroplast damage and cell death at low temperature. Plant J. 75, 539–552. doi: 10.1111/tpj.12219 23617639

[B132] Zhang C. Sui-zhang F. Arora R. Hannapel D. J. (2010). Ice recrystallization inhibition proteins of perennial ryegrass enhance freezing tolerance. Planta 232, 155–164. doi: 10.1007/s00425-010-1163-4 20379831

[B133] Zhang H. Zhang J. Xu Q. Wang D. Di H. Huang J.. (2020). Identification of candidate tolerance genes to low-temperature during maize germination by GWAS and RNA-seqapproaches. BMC Plant Biol. 20, 1–17. doi: 10.1186/S12870-020-02543-9/FIGURES/6 32664856 PMC7362524

[B134] Zhen Y. Ungerer M. C. (2008). Clinal variation in freezing tolerance among natural accessions of arabidopsis thaliana. New Phytol. 177, 419–427. doi: 10.1111/j.1469-8137.2007.02262.x 17995917

