# Supplementary material for: Identification of multiple novel genetic mechanisms that regulate chilling tolerance in Arabidopsis
Source: Front Plant Sci. 2023 Jan 12;13:1094462. doi: 10.3389/fpls.2022.1094462 (PMC9878698; doi:10.3389/fpls.2022.1094462)
Supplement: Supplementary file 1 [file DataSheet_1.docx]

**Figure S1. (a)** Geographical locations of 417 Arabidopsis ecotypes used in this study.

**(b)** QQ Plot of observed versus expected *p*-values for the changes of leaf area under prolonged cold stress GWAS analysis for all SNPs.

**(c)** Frequency distribution of the 417 accessions for proportionate cold tolerance. The location of reference accession Col-0 is indicated with a red arrow. Growth rate data for each accession are given in Table S2. Growth rate of each ecotype (%) is calculated as growth at termination of exposure to cold stress (on 30th day of treatment) X 100/Growth before initiation of treatment (0th day of treatment). Proportionate tolerance of each ecotype is calculated as growth rate of each ecotype X 100/ the summation of growth rates of 417 ecotypes (detailed information is on Supplemental Fig. S2).
