# Supplementary material for: Identification of multiple novel genetic mechanisms that regulate chilling tolerance in Arabidopsis
Source: Front Plant Sci. 2023 Jan 12;13:1094462. doi: 10.3389/fpls.2022.1094462 (PMC9878698; doi:10.3389/fpls.2022.1094462)
Supplement: Supplementary file 3 [file DataSheet_3.docx]

**Figure S3.** Expression patterns of sixteen identified cold responsive genes **(a-p)** during exposure to cold stress using the eFP database (Winter et al., 2007; <http://bar.utoronto.ca/efp2/Arabidopsis/Arabidopsis_eFPBrowser2.html>).
