# Supplementary material for: Identification of multiple novel genetic mechanisms that regulate chilling tolerance in Arabidopsis
Source: Front Plant Sci. 2023 Jan 12;13:1094462. doi: 10.3389/fpls.2022.1094462 (PMC9878698; doi:10.3389/fpls.2022.1094462)
Supplement: Supplementary file 4 [file DataSheet_4.docx]

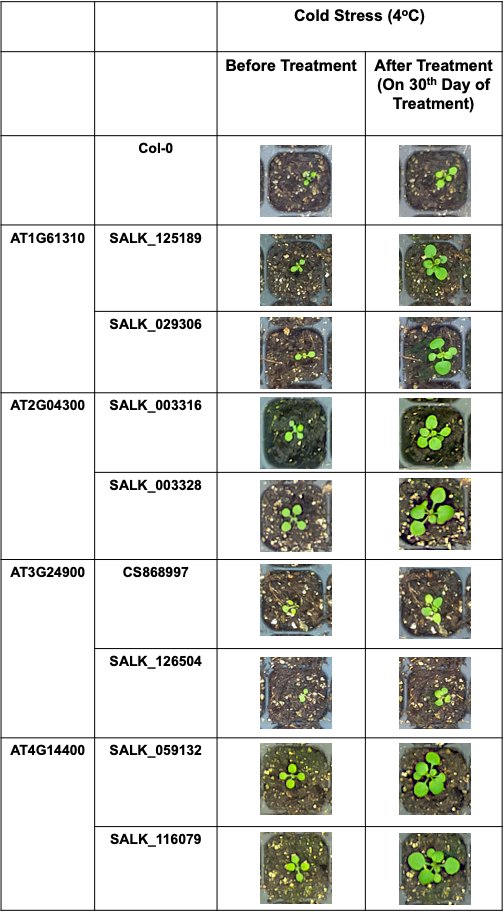


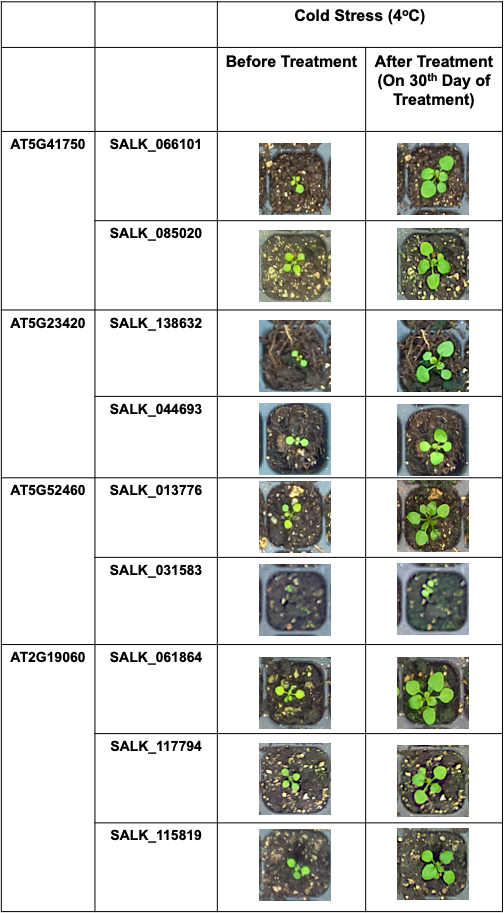


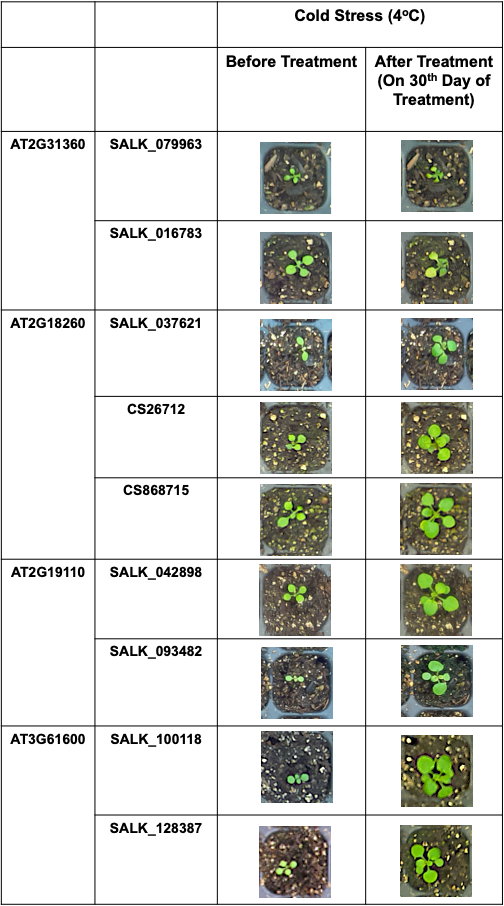


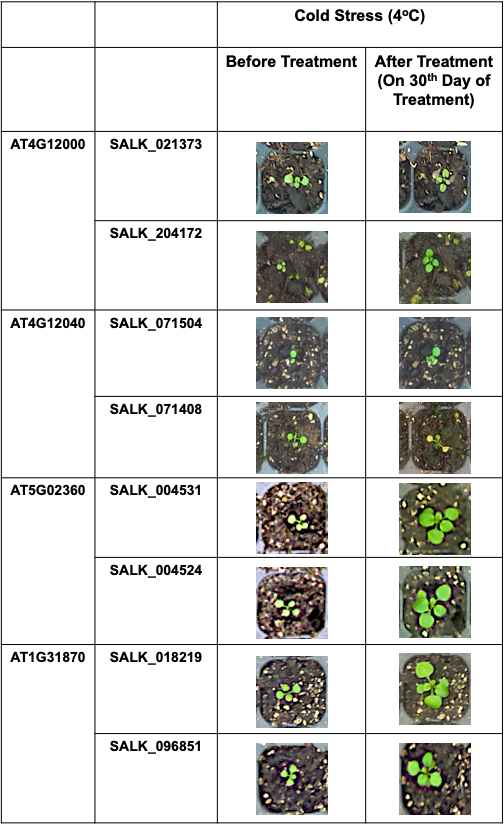


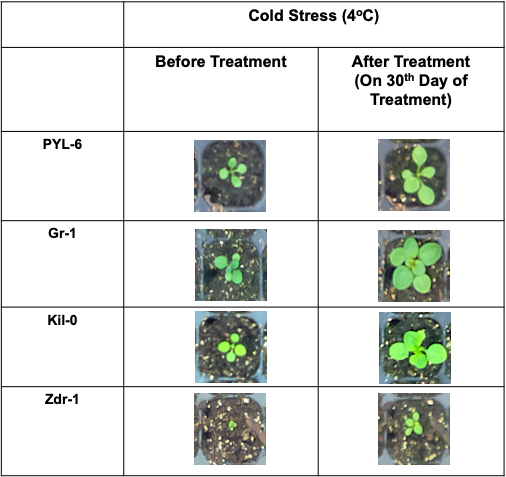


**Figure S4.** Differential tolerance of T-DNA mutants of the identified genes to continuous cold stress. Few representative cold-tolerant (PYL-6, Gr-1 and Kil-0) and cold-sensitive (Zdr-1) ecotypes are shown here.
