# Supplementary material for: Identification of multiple novel genetic mechanisms that regulate chilling tolerance in Arabidopsis
Source: Front Plant Sci. 2023 Jan 12;13:1094462. doi: 10.3389/fpls.2022.1094462 (PMC9878698; doi:10.3389/fpls.2022.1094462)
Supplement: Supplementary file 5 [file DataSheet_5.docx]

**Figure S5.** Nine regions of interest containing genes that contribute towards cold tolerance. **(a–i)** Each panel shows data for a genomic region of interest for which the mutant analysis uncovered cold stress responsive genes. On the left, output plot of *p*-values (−log base 10) in a 5-kb window for association of SNPs with phenotypic variation, obtained from easyGWAS is presented. On the right, rosette leaf growth rates of mutants with respect to Col-0 are presented. The relative rosette leaf growth rate in the mutant compared to wild-type Col-0 is significantly different in control (C) and cold stress (CS) (*p* < Bonferroni adjusted α) conditions. C, Comparative growth rate of the mutant relative to wild-type Col-0 in control condition; CS, Comparative growth rate of the mutant relative to wild-type Col-0 in cold stress.
