# Supplementary material for: Identification of multiple novel genetic mechanisms that regulate chilling tolerance in Arabidopsis
Source: Front Plant Sci. 2023 Jan 12;13:1094462. doi: 10.3389/fpls.2022.1094462 (PMC9878698; doi:10.3389/fpls.2022.1094462)
Supplement: Supplementary file 6 [file DataSheet_6.docx]

**Figure S6.** Gene Ontology (GO) analysis. The pie graphs showing grouping of 16 cold-response genes to (a) 58 classes based biological processes, (b) 17 classes based on molecular functions and (c) 13 classes based on their sub-cellular locations or as cellular components. The numbers in parentheses show the percentage of total genes in each functional categorization of genes. The summary of genes in each functional categorization is represented in Table S8-S10.
