# Supplementary material for: Identification of multiple novel genetic mechanisms that regulate chilling tolerance in Arabidopsis
Source: Front Plant Sci. 2023 Jan 12;13:1094462. doi: 10.3389/fpls.2022.1094462 (PMC9878698; doi:10.3389/fpls.2022.1094462)
Supplement: Supplementary file 7 [file DataSheet_7.docx]

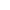


(a)


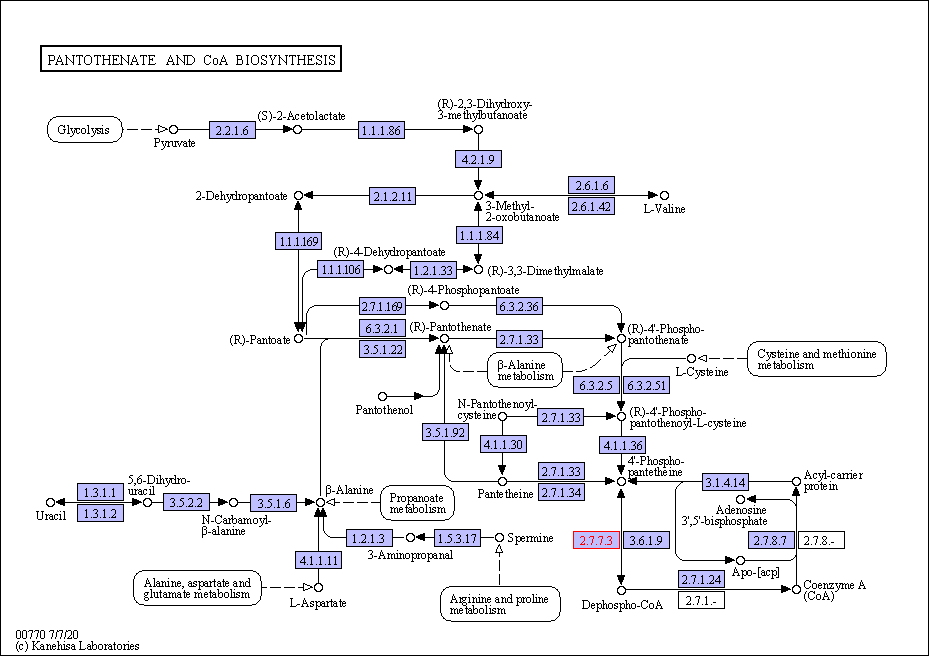


(b)


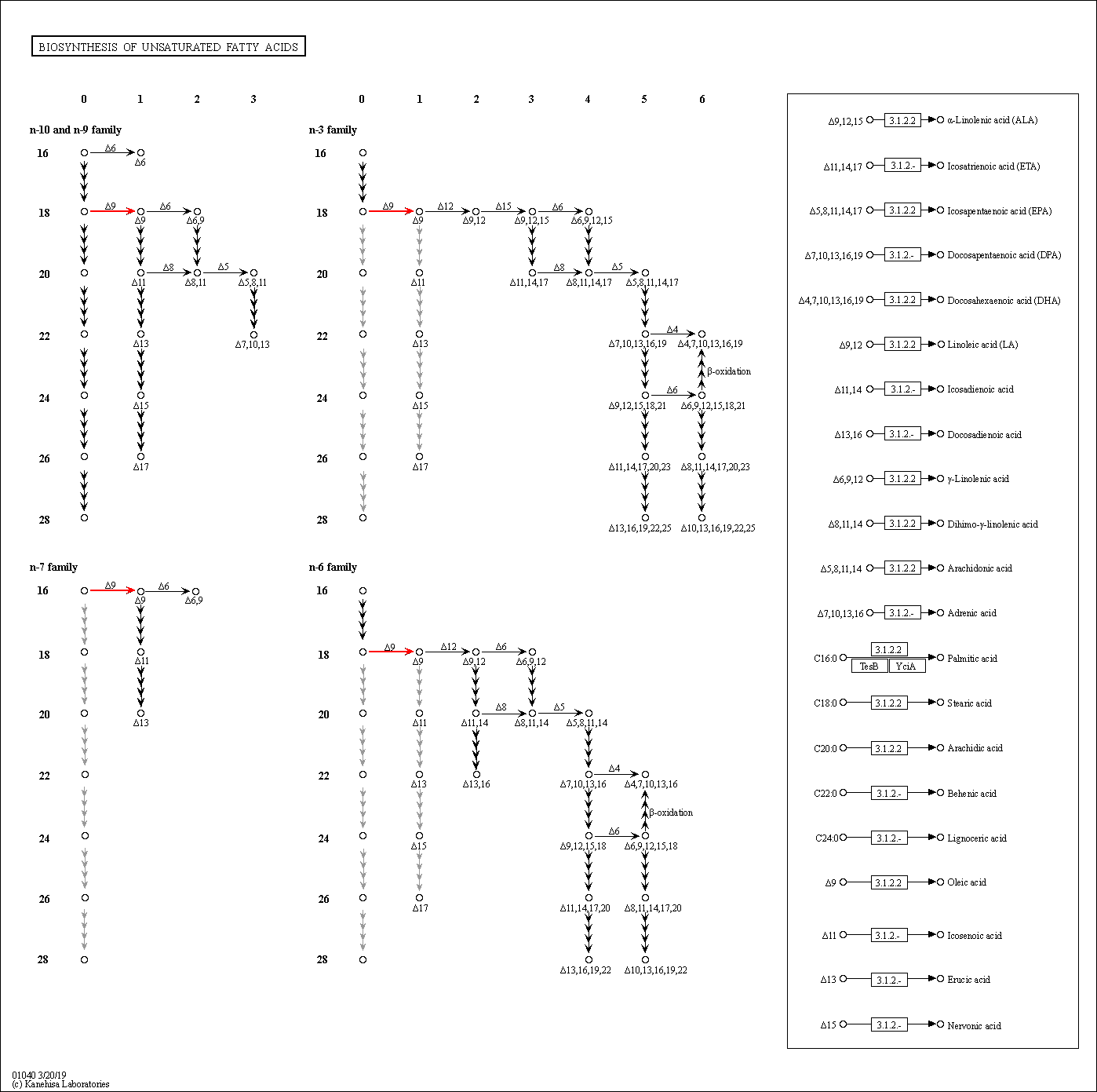


(c)


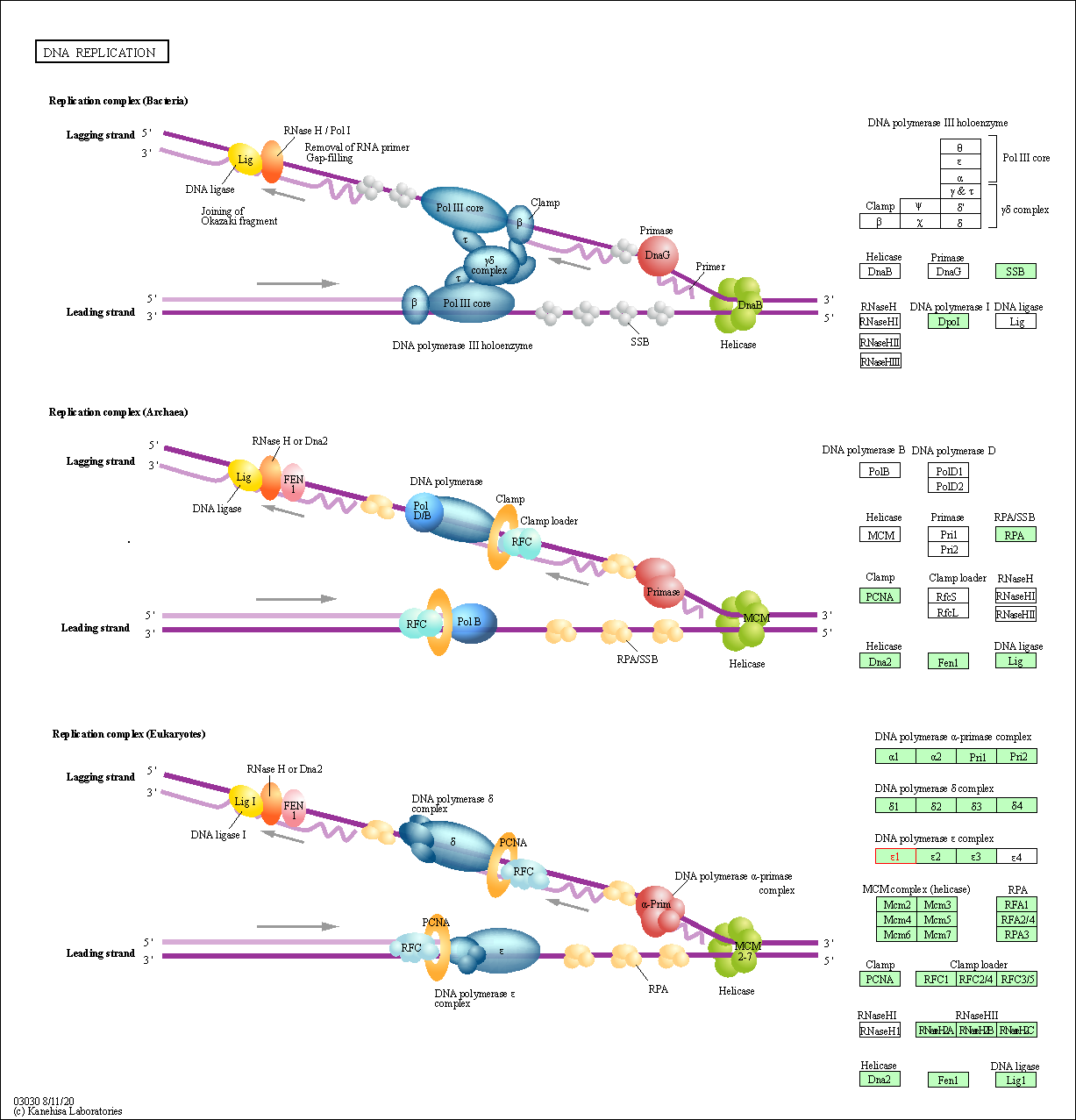


(d)


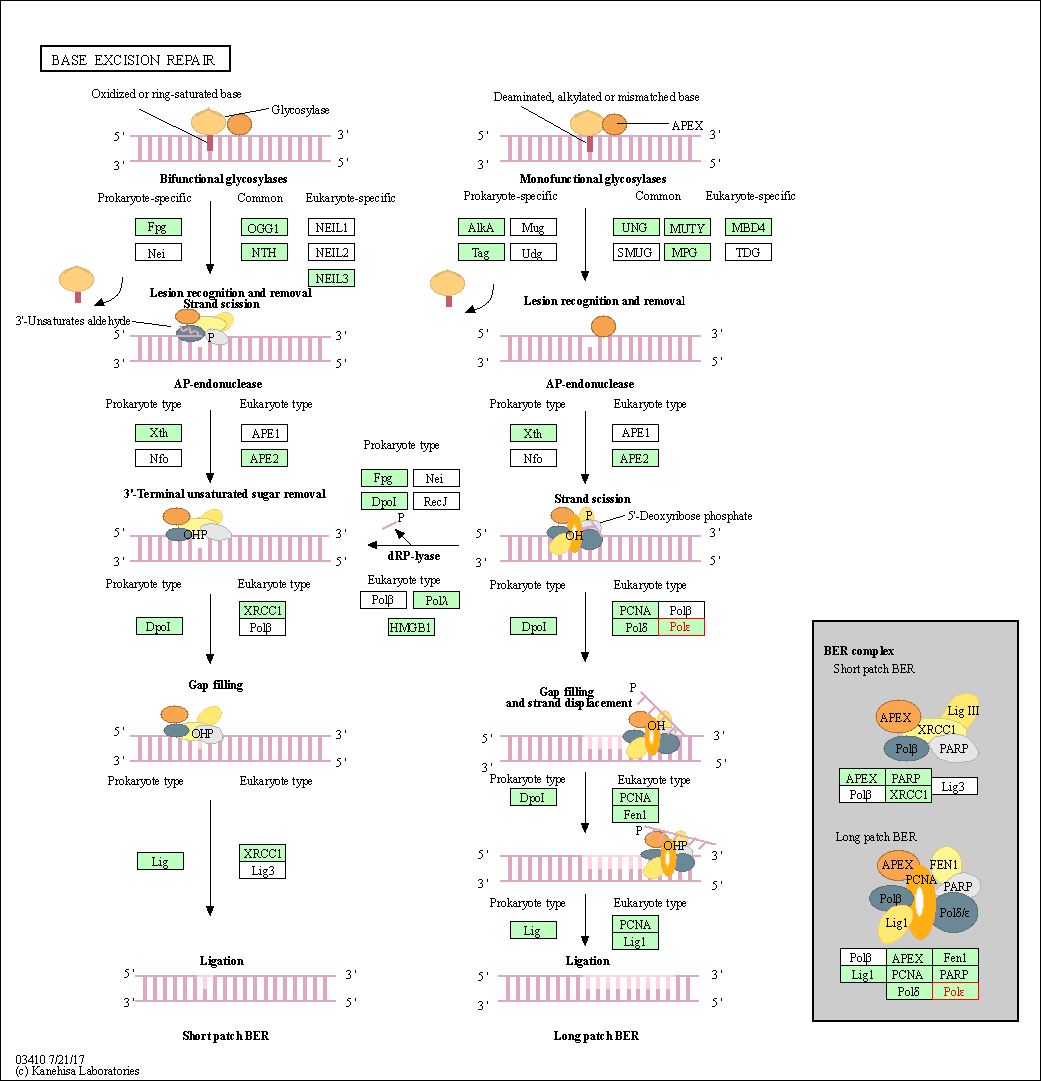


(e)


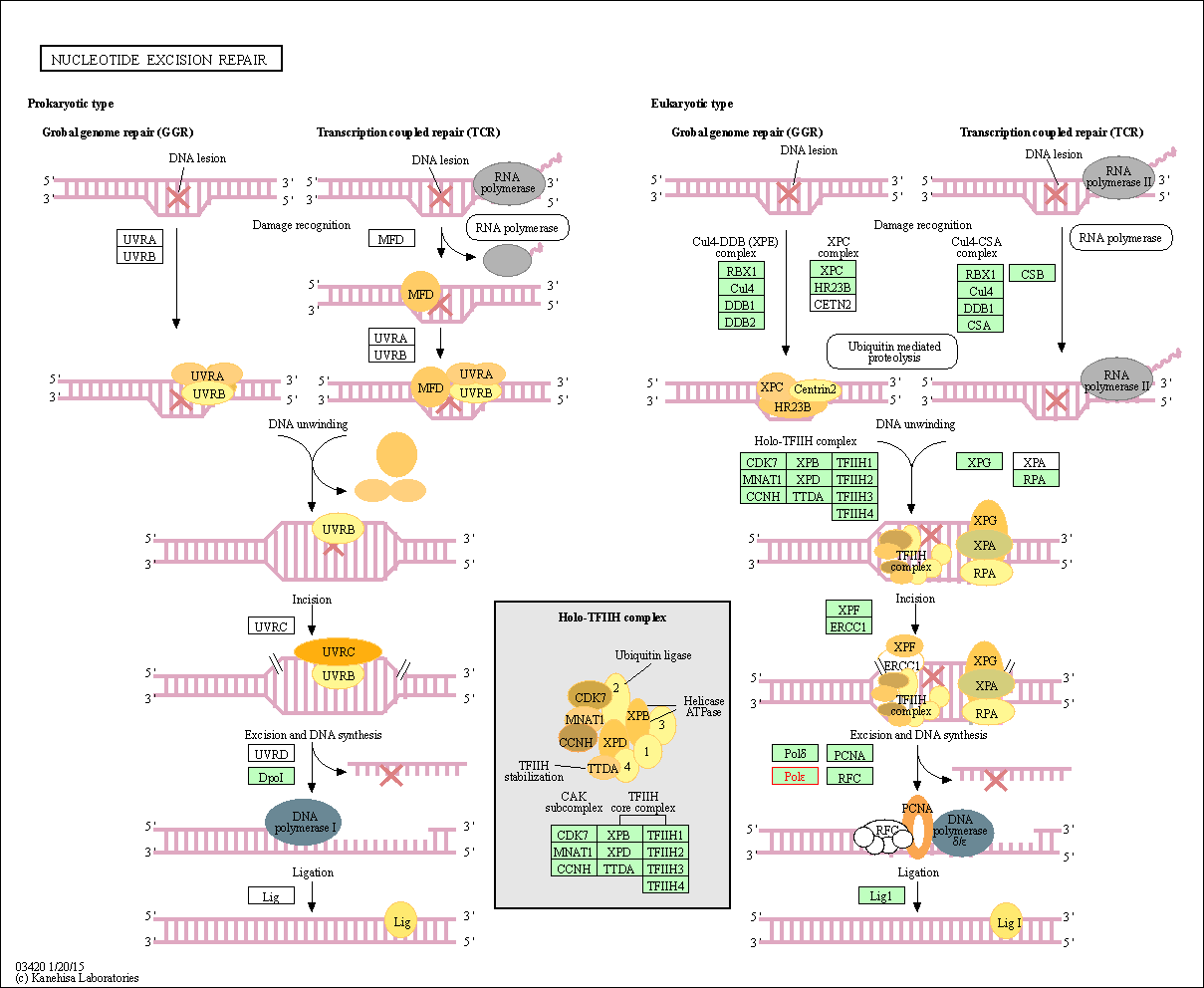


(f)


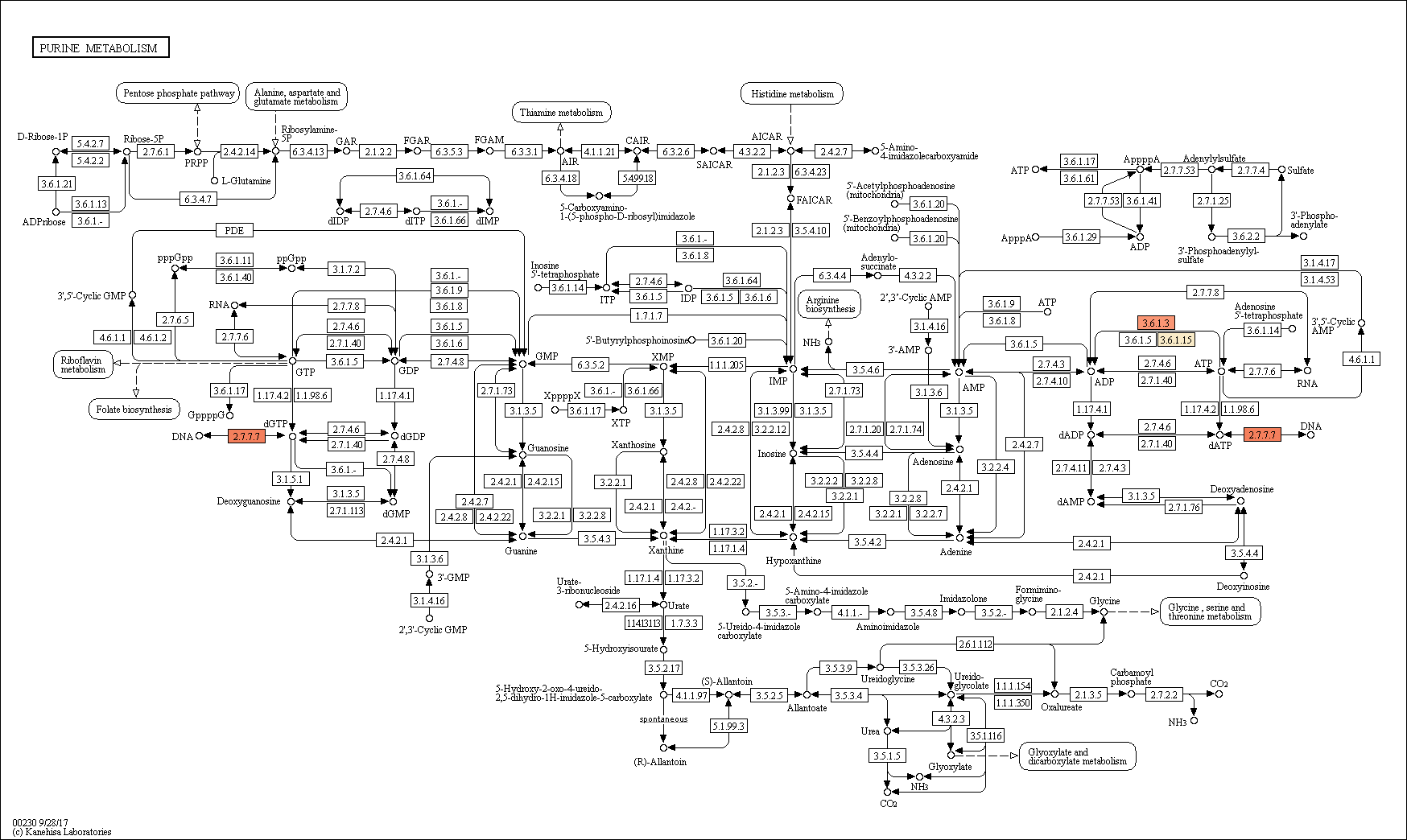


(g)


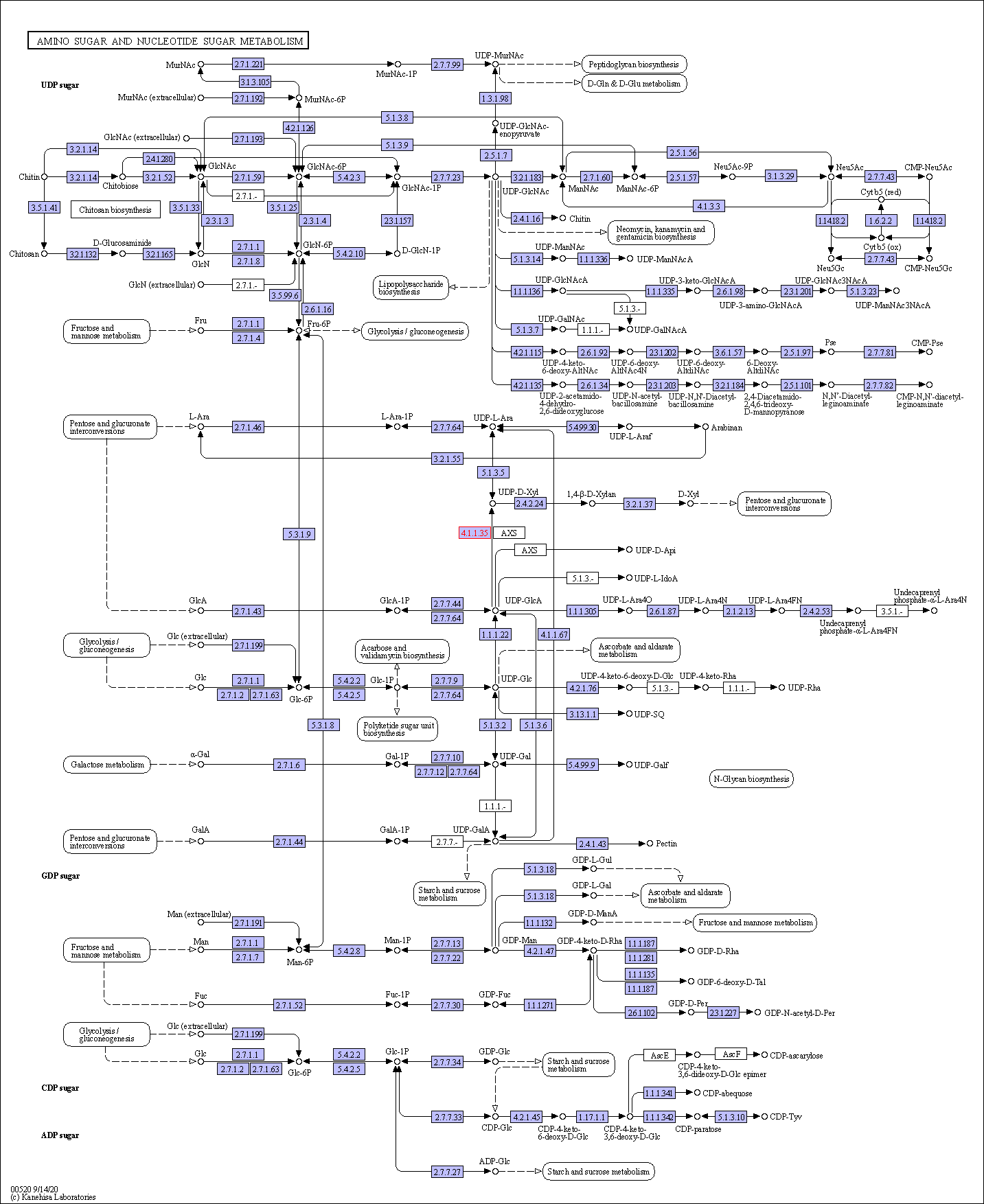


(h)


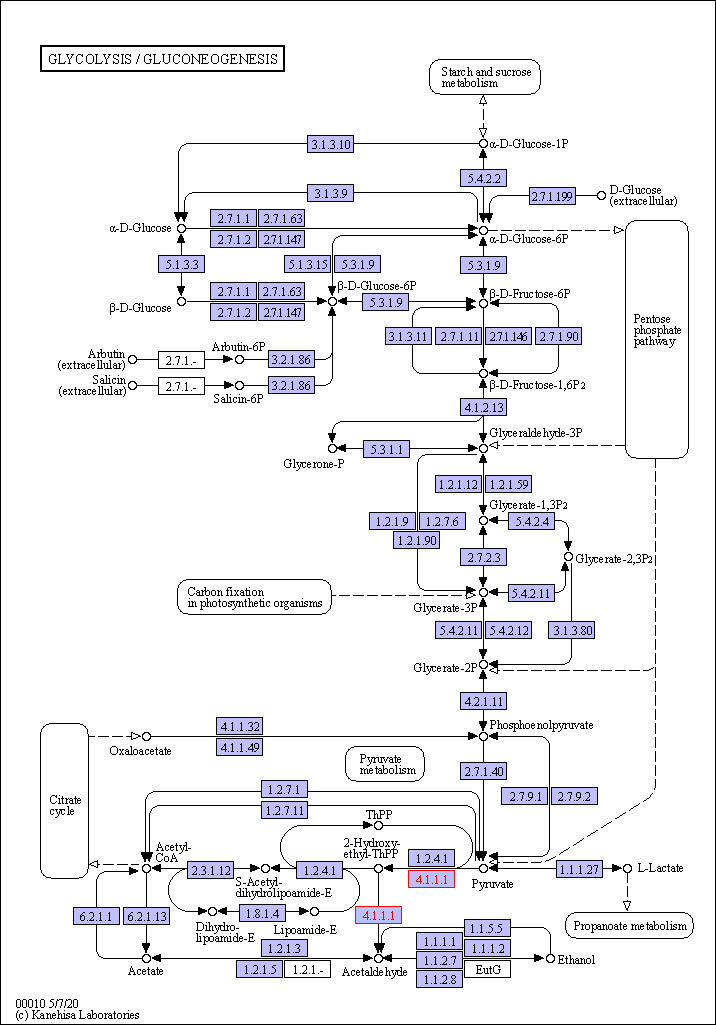


**Figure S7.** The KEGG pathways showing involvement of *AT2G18260* (EC:2.7.7.3) in pantothenate and CoA biosynthesis pathway (a), *AT2G31360* (EC:1.14.19.1) in biosynthesis of unsaturated fatty acids (b), *AT2G27120* (EC:2.7.7.7) in DNA replication, base/nucleotide excision repair and purine metabolism pathways (c-f), *AT3G53520* (EC:4.1.1.35) involved in amino sugar and nucleotide sugar metabolism pathways (g) and *AT5G54960* (EC:4.1.1.1) involved in glycolysis/gluconeogenesis (h).
