# Supplementary material for: Identification of multiple novel genetic mechanisms that regulate chilling tolerance in Arabidopsis
Source: Front Plant Sci. 2023 Jan 12;13:1094462. doi: 10.3389/fpls.2022.1094462 (PMC9878698; doi:10.3389/fpls.2022.1094462)
Supplement: Supplementary file 8 [file DataSheet_8.docx]

**A**

**B**

**C**

**D**

**E**

**F**

**G**

**H**

**I**

**Figure S8:**Overview of differentially regulated genes involved in different metabolic processes. Gene transcripts that are induced or repressed as a result of cold stress are shown for (a) metabolism overview, (b) biotic stress, (c) cellular response overview, (d) ubiquitin and autophagy-dependent degradation, (e) cell functions overview, (f) large enzyme families overview, (g) cell wall precursors, (h) Glycolysis-TCA, (i) RNA-protein synthesis. The figure was generated using the MapMan visualization tool (Usadel et al., 2009) on genes for which differential expression values were available (Tables 1,2; Fig. S3; Table S5).
