# Supplementary material for: Identification of multiple novel genetic mechanisms that regulate chilling tolerance in Arabidopsis
Source: Front Plant Sci. 2023 Jan 12;13:1094462. doi: 10.3389/fpls.2022.1094462 (PMC9878698; doi:10.3389/fpls.2022.1094462)
Supplement: Supplementary file 9 [file DataSheet_9.docx]

clc

source_folder = '../../data/';

dest_folder = '../../data/';

imname = 'T-11440';

%fname = [imname, num2str(imnum), '.png'];

num_cells = [12 12];

physical_size = [259 259];

cell_siz = 150;

imopts.num_cells = num_cells;

imopts.method = 'manual';

imopts.regtype = 'affine';

imopts.cell_siz = cell_siz;

imopts.display = 0;

imopts.siz = physical_size;

for imnum=43:70

fname = [source_folder, imname, num2str(imnum), '.png'];

im_orig = imread(fname);

im_reg = register_image(im_orig,imopts);

figure(1), clf

imshow(im_reg)

pause(0.5)

imwrite(im_reg,[dest_folder,'OutputImg',num2str(imnum),'.png'])

end
