# Supplementary material for: Identification of multiple novel genetic mechanisms that regulate chilling tolerance in Arabidopsis
Source: Front Plant Sci. 2023 Jan 12;13:1094462. doi: 10.3389/fpls.2022.1094462 (PMC9878698; doi:10.3389/fpls.2022.1094462)
Supplement: Supplementary file 10 [file DataSheet_10.docx]

close all

clc

% read

%imname = '../../data/ATCol-';

imname = '../../data/T-';

imnum = 43437;

%fname = [imname, num2str(imnum), '.jpg'];

fname = [imname, num2str(imnum), '.png'];

im_orig = imread(fname);

num_cells = [12 12];

physical_size = [259 259];

cell_siz = 150;

imopts.num_cells = num_cells;

imopts.method = 'auto';

imopts.regtype = 'projective';

imopts.cell_siz = cell_siz;

imopts.display = 1;

imopts.hsv_greenfilt = [0.18 0.4; 0.3 0.9; 0.75 1];

imopts.siz = physical_size;

% crop and register

im_reg = register_image(im_orig,imopts);

%return

% segment

im_cells = segment_image(im_reg,imopts);

% count leaf area

[leaf_areas,leaf_areas_pixels] = count_leaf_area(im_cells,imopts);

disp(leaf_areas)

% organize data, output

write_data = 1;

if write_data

csv_fname = ['pixelareas-', num2str(imnum), '.csv'];

dlmwrite(csv_fname,leaf_areas,'precision',4);

end
