# Supplementary material for: Identification of multiple novel genetic mechanisms that regulate chilling tolerance in Arabidopsis
Source: Front Plant Sci. 2023 Jan 12;13:1094462. doi: 10.3389/fpls.2022.1094462 (PMC9878698; doi:10.3389/fpls.2022.1094462)
Supplement: Supplementary file 18 [file Table_8.docx]

**Table S8.** Gene Ontology (GO) annotations for Biological process.

| GO-term | #Seqs | Gene ID |
| --- | --- | --- |
| response to organic substance | 1 | AT4G14400.1 |
| vesicle docking | 1 | AT2G18260.1 |
| cellular macromolecule localization | 1 | AT2G18260.1 |
| ion transmembrane transport | 1 | AT2G19110.1 |
| vesicle fusion | 1 | AT2G18260.1 |
| homeostatic process | 1 | AT2G19110.1 |
| response to cold | 2 | AT2G18260.1, AT4G14400.1 |
| response to oxygen-containing compound | 1 | AT4G14400.1 |
| organic cyclic compound metabolic process | 1 | AT1G31870.1 |
| regulation of signal transduction | 1 | AT4G14400.1 |
| secretion by cell | 1 | AT2G18260.1 |
| establishment of localization in cell | 1 | AT2G18260.1 |
| response to bacterium | 1 | AT4G14400.1 |
| response to oxidative stress | 1 | AT4G12000.1 |
| protein localization | 1 | AT2G18260.1 |
| salicylic acid mediated signaling pathway | 1 | AT4G14400.1 |
| cellular aromatic compound metabolic process | 1 | AT1G31870.1 |
| macromolecule metabolic process | 5 | AT3G61600.1, AT4G14400.1, AT4G12040.1, AT1G31870.1, AT2G04300.1 |
| nucleobase-containing compound metabolic process | 1 | AT1G31870.1 |
| organic acid metabolic process | 1 | AT2G31360.1 |
| regulation of response to external stimulus | 1 | AT4G14400.1 |
| response to inorganic substance | 1 | AT2G19110.1 |
| response to fungus | 1 | AT4G14400.1 |
| defense response to other organism | 1 | AT4G14400.1 |
| protein metabolic process | 4 | AT3G61600.1, AT4G14400.1, AT4G12040.1, AT2G04300.1 |
| cellular component organization | 1 | AT2G18260.1 |
| response to external biotic stimulus | 1 | AT4G14400.1 |
| positive regulation of response to external stimulus | 1 | AT4G14400.1 |
| cellular biosynthetic process | 1 | AT2G31360.1 |
| positive regulation of defense response | 1 | AT4G14400.1 |
| defense response | 2 | AT1G61310.1, AT4G14400.1 |
| positive regulation of response to biotic stimulus | 1 | AT4G14400.1 |
| response to virus | 1 | AT4G14400.1 |
| regulation of cell communication | 1 | AT4G14400.1 |
| intracellular transport | 1 | AT2G18260.1 |
| organic substance catabolic process | 2 | AT2G19060.1, AT4G12040.1 |
| cellular nitrogen compound metabolic process | 1 | AT1G31870.1 |
| organonitrogen compound metabolic process | 4 | AT3G61600.1, AT4G14400.1, AT4G12040.1, AT2G04300.1 |
| response to temperature stimulus | 2 | AT2G18260.1, AT4G14400.1 |
| innate immune response | 1 | AT4G14400.1 |
| establishment of protein localization | 1 | AT2G18260.1 |
| heterocycle metabolic process | 1 | AT1G31870.1 |
| cellular lipid metabolic process | 1 | AT2G31360.1 |
| cellular response to chemical stimulus | 1 | AT4G14400.1 |
| cellular catabolic process | 1 | AT4G12040.1 |
| organelle localization | 1 | AT2G18260.1 |
| small molecule biosynthetic process | 1 | AT2G31360.1 |
| cellular macromolecule metabolic process | 4 | AT3G61600.1, AT4G14400.1, AT4G12040.1, AT2G04300.1 |
| organelle localization by membrane tethering | 1 | AT2G18260.1 |
| transport | 2 | AT2G18260.1, AT2G19110.1 |
| response to oomycetes | 1 | AT4G14400.1 |
| organic substance biosynthetic process | 1 | AT2G31360.1 |
| regulation of response to stress | 1 | AT4G14400.1 |
| regulation of response to biotic stimulus | 1 | AT4G14400.1 |
| lipid metabolic process | 2 | AT2G19060.1, AT2G31360.1 |
| exocytosis | 1 | AT2G18260.1 |
| phosphorus metabolic process | 1 | AT2G04300.1 |
| response to radiation | 2 | AT3G61600.1, AT4G14400.1 |
