# Supplementary material for: Identification of multiple novel genetic mechanisms that regulate chilling tolerance in Arabidopsis
Source: Front Plant Sci. 2023 Jan 12;13:1094462. doi: 10.3389/fpls.2022.1094462 (PMC9878698; doi:10.3389/fpls.2022.1094462)
Supplement: Supplementary file 19 [file Table_9.docx]

**Table S9.** Gene Ontology (GO) annotations for Molecular Function.

| GO-term | #Seqs |  |
| --- | --- | --- |
| inorganic molecular entity transmembrane transporter activity | 1 | AT2G19110.1 |
| SNAP receptor activity | 1 | AT2G18260.1 |
| identical protein binding | 1 | AT3G61600.1 |
| protein kinase activity | 1 | AT2G04300.1 |
| nucleic acid binding | 2 | AT5G23420.1, AT4G12040.1 |
| SNARE binding | 1 | AT2G18260.1 |
| hydrolase activity, acting on ester bonds | 1 | AT2G19060.1 |
| active transmembrane transporter activity | 1 | AT2G19110.1 |
| nucleoside phosphate binding | 4 | AT2G19110.1, AT5G41750.1, AT1G61310.1, AT2G04300.1 |
| transferase activity, transferring phosphorus-containing groups | 1 | AT2G04300.1 |
| ribonucleotide binding | 4 | AT2G19110.1, AT5G41750.1, AT1G61310.1, AT2G04300.1 |
| ion transmembrane transporter activity | 1 | AT2G19110.1 |
| oxidoreductase activity, acting on paired donors, with incorporation or reduction of molecular oxygen | 1 | AT2G31360.1 |
| anion binding | 4 | AT2G19110.1, AT5G41750.1, AT1G61310.1, AT2G04300.1 |
| protein dimerization activity | 1 | AT3G61600.1 |
| cation binding | 2 | AT2G19110.1, AT4G12040.1 |
| nucleotide binding | 4 | AT2G19110.1, AT5G41750.1, AT1G61310.1, AT2G04300.1 |
