# Supplementary material for: Identification of multiple novel genetic mechanisms that regulate chilling tolerance in Arabidopsis
Source: Front Plant Sci. 2023 Jan 12;13:1094462. doi: 10.3389/fpls.2022.1094462 (PMC9878698; doi:10.3389/fpls.2022.1094462)
Supplement: Supplementary file 20 [file Table_10.docx]

**Table S10.** Gene Ontology (GO) annotations for Cellular Component.

| GO-term | #Seqs |  |
| --- | --- | --- |
| anchoring junction | 2 | AT2G19110.1, AT4G12040.1 |
| spliceosomal complex | 1 | AT1G31870.1 |
| plasma membrane | 4 | AT2G18260.1, AT2G19110.1, AT4G14400.1, AT2G04300.1 |
| intrinsic component of organelle membrane | 1 | AT4G14400.1 |
| intracellular membrane-bounded organelle | 6 | AT5G23420.1, AT2G31360.1, AT2G18260.1, AT3G61600.1, AT4G14400.1, AT1G31870.1 |
| nuclear outer membrane-endoplasmic reticulum membrane network | 2 | AT2G31360.1, AT4G14400.1 |
| intrinsic component of plasma membrane | 1 | AT4G14400.1 |
| integral component of membrane | 7 | AT2G31360.1, AT2G18260.1, AT2G19110.1, AT4G14400.1, AT2G04300.1, AT3G24900.1, AT4G12000.1 |
| membrane-bounded organelle | 6 | AT2G31360.1, AT5G23420.1, AT2G18260.1, AT3G61600.1, AT4G14400.1, AT1G31870.1 |
| plasmodesma | 2 | AT2G19110.1, AT4G12040.1 |
| SNARE complex | 1 | AT2G18260.1 |
| organelle membrane | 2 | AT2G31360.1, AT4G14400.1 |
| endoplasmic reticulum | 2 | AT2G31360.1, AT4G14400.1 |
